# Specific Roles of XRCC4 Paralogs PAXX and XLF during V(D)J Recombination

**DOI:** 10.1016/j.celrep.2016.08.069

**Published:** 2016-09-02

**Authors:** Chloé Lescale, Hélène Lenden Hasse, Andrew N. Blackford, Gabriel Balmus, Joy J. Bianchi, Wei Yu, Léa Bacoccina, Angélique Jarade, Christophe Clouin, Rohan Sivapalan, Bernardo Reina-San-Martin, Stephen P. Jackson, Ludovic Deriano

**Affiliations:** 1 Departments of Immunology and Genomes and Genetics, Institut Pasteur, 75015 Paris, France; 2 Weatherall Institute of Molecular Medicine, University of Oxford, John Radcliffe Hospital, Oxford OX3 9DS, UK; 3 CRUK/MRC Oxford Institute for Radiation Oncology, Department of Oncology, University of Oxford, Oxford OX3 7DQ, UK; 4 The Wellcome Trust and Cancer Research UK Gurdon Institute, University of Cambridge, Cambridge CB2 1QN, UK; 5 Maintenance of Genome Stability, Genome Campus, Wellcome Trust Sanger Institute, Cambridge CB10 1SA, UK; 6 Cellule Pasteur, University of Paris René Descartes, Sorbonne Paris Cité, Paris 75015, France; 7 Institut de Génétique et de Biologie Moléculaire et Cellulaire, INSERM-U964, CNRS-UMR7104, University of Strasbourg, 67400 Illkirch, France

**Keywords:** V(D)J recombination, DNA repair, NHEJ, PAXX, XLF, XRCC4

## Abstract

Paralog of XRCC4 and XLF (PAXX) is a member of the XRCC4 superfamily and plays a role in nonhomologous end-joining (NHEJ), a DNA repair pathway critical for lymphocyte antigen receptor gene assembly. Here, we find that the functions of PAXX and XLF in V(D)J recombination are masked by redundant joining activities. Thus, combined PAXX and XLF deficiency leads to an inability to join RAG-cleaved DNA ends. Additionally, we demonstrate that PAXX function in V(D)J recombination depends on its interaction with Ku. Importantly, we show that, unlike XLF, the role of PAXX during the repair of DNA breaks does not overlap with ATM and the RAG complex. Our findings illuminate the role of PAXX in V(D)J recombination and support a model in which PAXX and XLF function during NHEJ repair of DNA breaks, whereas XLF, the RAG complex, and the ATM-dependent DNA damage response promote end joining by stabilizing DNA ends.

## Highlights

• PAXX-deficient pro-B cells support normal V(D)J recombination
• PAXX and XLF are mutually redundant in repairing RAG-DNA breaks
• PAXX function in V(D)J recombination depends on its interaction with Ku
• Unlike XLF, PAXX is not redundant with ATM and the RAG complex in repairing DNA breaks

## Graphical Abstract

**Figure:**
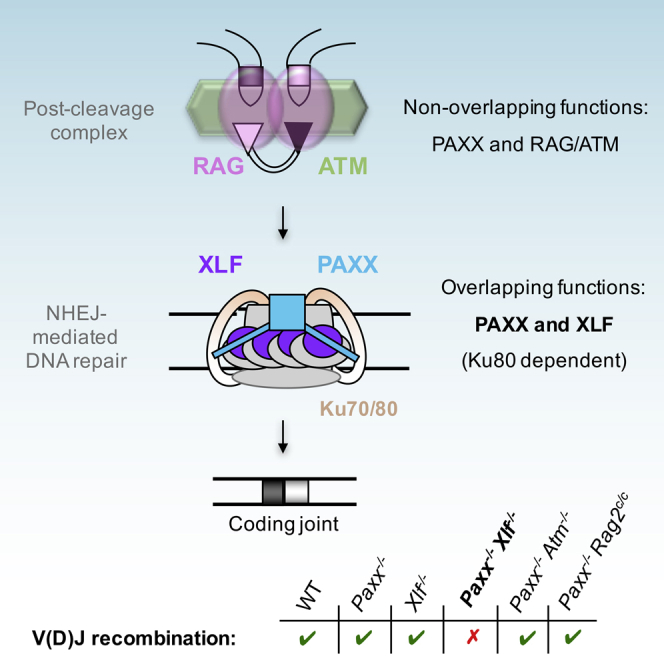

## Teaser

Developing lymphocytes rely on nonhomologous end joining (NHEJ) to repair programmed DNA double-strand breaks generated during antigen receptor gene assembly. Lescale et al. show that PAXX—a component of the NHEJ machinery—has a key role in V(D)J recombination that is masked by functional redundancy with its paralog XLF.

## Introduction

V(D)J recombination assembles immunoglobulin and T cell receptor variable exons from variable (V), diversity (D), and joining (J) gene segments via a cut-and-paste mechanism (Bassing et al., 2002). This process occurs in developing lymphocytes during the G1 phase of the cell cycle and is initiated when the recombination-activating gene products RAG1 and RAG2 (forming the RAG endonuclease) introduce double-strand breaks (DSBs) among V, D, and J coding gene segments and flanking recombination signal sequences (RSSs) (Schatz and Swanson, 2011). RAG-mediated cleavage at a pair of RSSs generates four broken DNA ends: two blunt 5′ phosphorylated signal ends (SEs) that terminate in the RSS and two covalently sealed (hairpin) coding ends (CEs). After cleavage, the RAG proteins stay associated with the DNA ends in a post-cleavage complex (PCC) that is thought to contribute to end-stabilization and end-joining activities (Deriano and Roth, 2013, Schatz and Swanson, 2011). Subsequently, the classical nonhomologous end-joining (NHEJ) pathway joins these DNA ends in a recombinant configuration, forming a coding joint (CJ) (the rearranged antigen receptor gene) and a reciprocal signal joint (SJ) (Deriano and Roth, 2013, Helmink and Sleckman, 2012, Schatz and Swanson, 2011). Additionally, RAG-induced DNA breaks activate the ataxia telangiectasia mutated (ATM)-kinase-dependent DNA damage response (DDR) (Helmink and Sleckman, 2012). ATM, beyond activating p53-dependent G1/S checkpoints, contributes to the repair of chromosomal DSBs by stabilizing coding ends in post-cleavage repair complexes most likely through the activation of downstream targets (Bredemeyer et al., 2006). Thus, the stabilization and tethering of broken DNA ends depends on ATM kinase activity and the formation of ATM-dependent DNA repair foci (Helmink and Sleckman, 2012, Kumar et al., 2014).

During NHEJ, the Ku70/80 heterodimer (Ku) binds DNA ends and recruits the DNA-dependent protein kinase catalytic subunit (DNA-PKcs) in order to form the DNA-PK holoenzyme (Gottlieb and Jackson, 1993). DNA-PK phosphorylates multiple substrates, promoting synapsis of DNA ends and facilitating the recruitment of end processing and ligation enzymes. Finally, the XRCC4-Ligase 4 complex performs ligation of DNA ends (Deriano and Roth, 2013, Lieber, 2010). Deficiency for these core NHEJ factors results in severe combined immunodeficiency due to the inability to complete repair of RAG DNA breaks (Blunt et al., 1995, Deriano and Roth, 2013, Helmink and Sleckman, 2012, Revy et al., 2005, Rooney et al., 2004), thus highlighting the importance of identifying all players in this process and deciphering their functions.

XRCC4 and the XRCC4-like factor (XLF) are two members of the same protein family and share structural similarity (Andres et al., 2007, Callebaut et al., 2006, Li et al., 2008b). XLF stimulates the XRCC4/Ligase 4 complex through an uncertain mechanism, and together, XLF and XRCC4 form long filaments thought to help DNA end tethering during NHEJ (Hammel et al., 2011, Mahaney et al., 2013, Reid et al., 2015, Riballo et al., 2009, Ropars et al., 2011, Roy et al., 2015, Tsai et al., 2007). In contrast to other NHEJ factor deficiencies, XLF-deficient mice are not markedly immune-deficient, and pro-B cell lines derived from these animals perform nearly normal V(D)J recombination (Lescale and Deriano, 2016, Li et al., 2008a, Vera et al., 2013). This apparently nonessential role is at least partly due to functional redundancy between XLF and members of the ATM DDR (Kumar et al., 2014, Zha et al., 2011). Additionally, we recently demonstrated that XLF function in the repair of RAG DNA breaks is also masked by redundancy with RAG2 (Lescale et al., 2016). Consistent with this, induction of RAG-mediated recombination in pro-B cell lines deficient for XLF and ATM or expressing a C-terminal truncated form of RAG2 in the absence of XLF similarly leads to a block in V(D)J recombination and severe immune-deficiency due to an inability to repair DSB intermediates (Kumar et al., 2014, Lescale et al., 2016, Zha et al., 2011).

PAXX, a paralog of XRCC4 and XLF (Ochi et al., 2015), is a recently identified component of the NHEJ machinery (Craxton et al., 2015, Ochi et al., 2015, Xing et al., 2015). It has been shown to accumulate at sites of DNA damage and function with XRCC4 and XLF to mediate DSB repair and cell survival in response to DSB-inducing agents. PAXX itself does not bind DNA but interacts with Ku and Ku-bound DNA in order to promote NHEJ factor assembly and enhance DNA end ligation (Ochi et al., 2015, Roy et al., 2015, Xing et al., 2015). In addition, episomal recombination assays performed in HEK293 cells showed that loss of PAXX accentuates the requirement for XLF in joining coding and signal ends (Roy et al., 2015), indicating that PAXX might play important functions during antigen receptor gene assembly in lymphocytes. Here, we used a physiological RAG-inducible B cell line system to investigate the role of PAXX during V(D)J recombination and uncover potential functional interactions with XLF, the ATM-dependent DDR and the RAG complex during repair of RAG-generated DSB intermediates.

## Results

### Generation of PAXX-Deficient *v-abl* pro-B Cells using CRISPR/Cas9 Gene Editing

**Figure 1:**
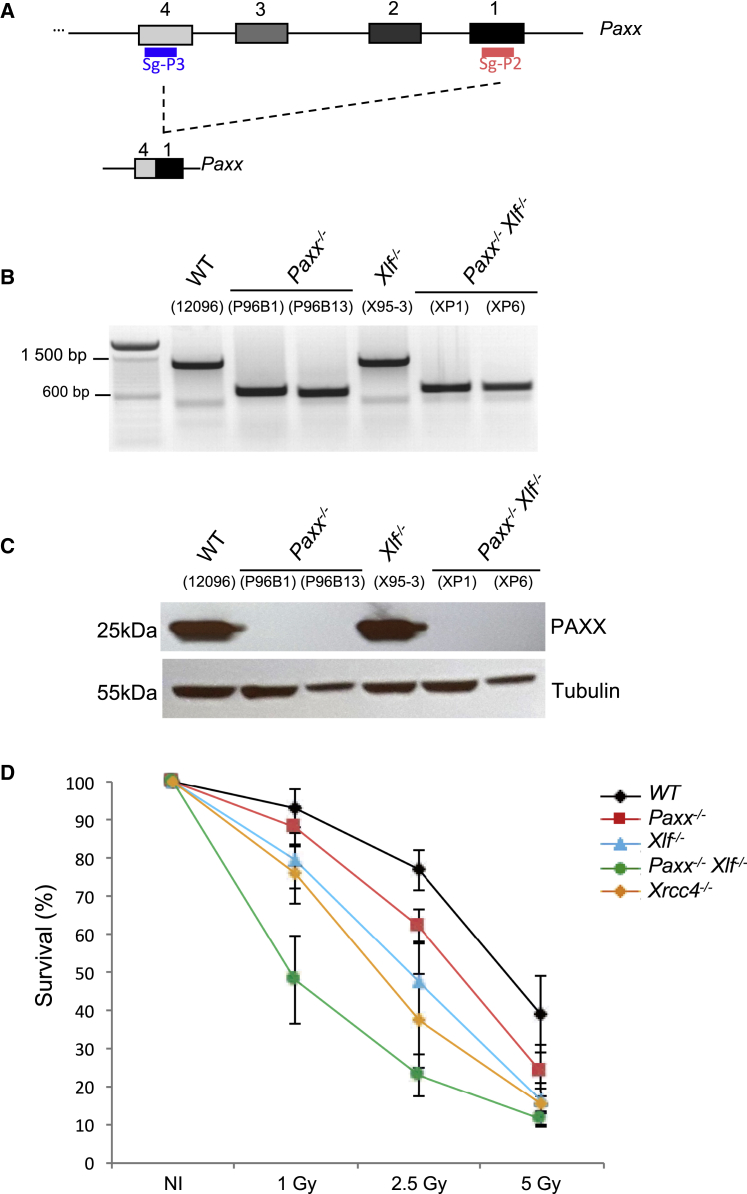
CRISPR/Cas9-Mediated Deletion of *Paxx* in *v-abl* pro-B Cells and Irradiation Sensitivity (A) CRISPR/Cas9 *Paxx* knockout strategy. Exons are shown in boxes, and sgRNA positions are indicated. See also Figure S1A. (B) PCR analysis showing the deletion of approximately 626 bp in the *Paxx* gene (WT = 1,299 bp band; Paxx KO ≈673 bp band). See also Figure S1B. (C) Western blot showing the absence of the PAXX protein in *Paxx*-deleted pro B cell clones. See also Figure S1C. (D) Irradiation sensitivity of *v-abl* pro-B cell lines. The graph represents mean ± SEM of three independent experiments using two independent cell lines (see also Table S2). Numbers indicate the percentage of survival 3 days after irradiation at 1, 2.5, and 5 Gy. See also Table S2.

We employed CRISPR/Cas9-mediated gene editing to delete *Paxx* (Δexons1–4) from wild-type (WT) v-Abelson (*v-abl*) immortalized pro-B cells (Lescale et al., 2016), generating *Paxx*^*−/−*^ *v-abl* pro-B cell clones (Figures 1A and S1A; Table S1). *Paxx*-deleted clones were selected on the basis of PCR analysis (Figures 1B and S1B) and complete loss of PAXX protein expression (Figures 1C and S1C). In addition, we generated *Xlf*^*−/−*^ and *Paxx*^*−/−*^ *Xlf*^*−/−*^ *v-abl* pro-B cell clones by deleting *Xlf* (Δexon1) from wild-type and *Paxx*^*−/−*^ cells (Figure S1A; Table S1; data not shown), respectively. We also generated *Xrcc4*^*−/−*^ clones by deleting exon3 of *Xrcc4*, which encodes part of the XRCC4 functional core region (Gao et al., 1998, Mizuta et al., 1997), from wild-type *v-abl* pro-B cells (Figure S1A; Table S1; data not shown). To test whether PAXX-deficient *v-abl* pro-B cells harbor defects in NHEJ-mediated DSB repair, we performed survival assays after exposing the cells to ionizing radiation. We found that *Paxx*^*−/−*^ *v-abl* pro-B cell clones were significantly more radiosensitive than wild-type *v-abl* pro-B cell lines but less sensitive than XLF- and XRCC4-deficient *v-abl* pro-B cells (Figure 1D; Table S2). Strikingly, the loss of both PAXX and XLF in *Paxx*^*−/−*^ *Xlf*^*−/−*^ pro-B cells led to extreme radiosensitivity in comparison to WT, PAXX, XLF, and XRCC4 single-mutant cells. Thus, we find that PAXX and XLF are not epistatic for the repair of irradiation-induced DNA damage in mouse *v-abl* pro-B cells.

### *Igk* Rearrangement in PAXX- and PAXX/XLF-Deficient pro-B Cells

**Figure 2:**
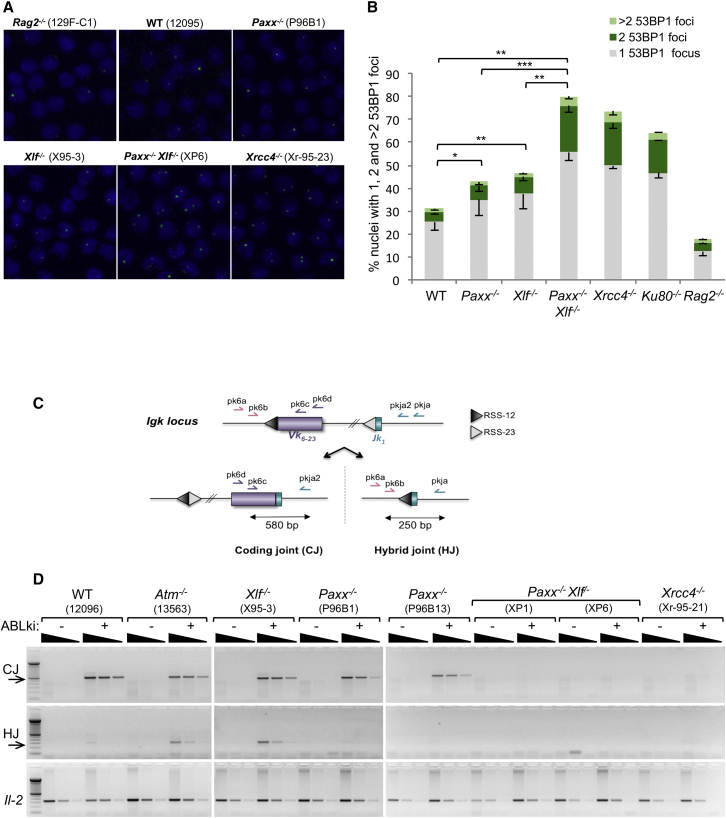
Accumulation of 53BP1 DDR Foci and Impaired *Igk* Rearrangement in *Paxx*^*−/−*^*Xlf*^*−/−*^ pro-B Cells (A) Representative 3D projections of 53BP1 immunostaining conducted on ABLki-treated *v-abl* pro–B cells. (B) Percentage of *v-abl* pro–B cells harboring 1, 2, or >2 53BP1 foci 65 hr post ABLki treatment. Data represent mean ± SEM from three independent experiments with one or two independent cell lines for each genotype. See also Table S3. (C) Schematic of the *Igk* locus with position of primers (arrows) used to assay coding joint (CJ) and hybrid joint (HJ) formation during inversional *IgkV*_*6-23*_*-J*_*1*_ rearrangement. (D) Semiquantitative nested PCR analysis of *IgkV*_*6-23*_*-J*_*1*_ coding joints (CJ) and hybrid joints (HJ) from indicated *v-abl* pro-B cell lines treated for 72h with ABLki. *Il-2* gene PCR was used as a loading control.

Treatment of *v-abl* immortalized pro-B cells with a *v-abl* kinase inhibitor (STI571, hereafter referred to as ABLki) leads to G1 cell-cycle arrest, the rapid induction and stabilization of RAG1/2 gene expression, and rearrangement of the endogenous *Igk* locus or any introduced V(D)J recombination reporter substrate (Bredemeyer et al., 2006, Lescale et al., 2016, Muljo and Schlissel, 2003). To elucidate whether PAXX has a role in RAG-mediated DSB repair in lymphocytes, we initially quantified the presence of DNA-damage-associated protein (53BP1) foci at the *Igk* locus in G1-arrested pro-B cells using automated 3D microscopy (Lescale et al., 2016). As expected (Lescale et al., 2016), upon treatment with ABLki for 3 days, we found that 31.1% of WT pro-B cells showed intense 53BP1 foci, the majority of which contained a single distinct spot, although cells were occasionally found to contain two, and less frequently three or more, foci (Figures 2A and 2B; Table S3). In ABLki-treated RAG2-deficient pro-B cells (*Rag2*^*−/−*^), 53BP1 foci were detected in only 18.2% of the cells, consistent with the absence of RAG cleavage in these cells (Lescale et al., 2016, Shinkai et al., 1992). The few foci observed in the absence of RAG activity likely result from incomplete DNA synthesis during S phase leading to the DDR and formation of 53BP1 domains during subsequent G1 (Harrigan et al., 2011). Interestingly, 43% of PAXX-deficient pro-B cells showed 53BP1 foci, slightly more than in WT pro-B cells and comparable to XLF-deficient cells (46.6%). However, strikingly, we found that 80.1% of *Paxx*^*−/−*^ *Xlf*^*−/−*^ pro-B cells harbored 53BP1 foci similar to Ku80 (64.5%) and XRCC4 (73.5%) deficiency (Figures 2A and 2B; Table S3). Also reminiscent of Ku80- and XRCC4-deficient pro-B cells, 20.1% of *Paxx*^*−/−*^ *Xlf*^*−/−*^ cells contained two 53BP1 foci corresponding to DNA breaks at both *Igk* alleles (Lescale et al., 2016) in comparison to 3.6%, 4.3%, 6.1%, and 7.3% in *Rag2*^*−/−*^, WT, *Paxx*^*−/−*^, and *Xlf*^*−/−*^ pro-B cells, respectively (Figures 2A and 2B; Table S3). These results indicate that RAG-mediated DNA breaks are readily formed in PAXX- and PAXX/XLF-deficient cells, however, and in contrast to single deficiency, repair of these DNA breaks does not seem to occur in combined PAXX/XLF deficient cells, paralleling what is seen in the absence of the canonical NHEJ factors Ku80 and XRCC4.

Consistent with the accumulation of 53BP1 foci in *Paxx*^*−/−*^ *Xlf*^*−/−*^ pro-B cells, PCR amplification of inversional *IgkV*_*6-23*_*-J*_*1*_ rearrangement (Figure 2C) in these cells revealed an almost complete lack of CJ formation, validating the presence of a specific end-joining defect in the absence of functional PAXX and XLF during V(D)J recombination (Figure 2D). Induction of RAG in WT, *Xlf*^*−/*^,^*−*^ and *Paxx*^*−/−*^ pro-B cells triggered robust *Vk*-to-*Jk* inversional CJ formation, whereas there was a complete absence of *IgkV*_*6-23*_*-J*_*1*_ rearrangements after the induction of RAG in *Xrcc4*^*−/−*^ pro-B cells due to the function of XRCC4 in repairing RAG-DSBs (Li et al., 1995). Notably, nested PCR amplification of inversional *IgkV*_*6-23*_*-J*_*1*_ rearrangement also revealed the formation of deletional hybrid joints (HJs), which results from the aberrant joining of a coding to a signal end, in *Atm*^*−/−*^ and in *Xlf*^*−/−*^ cells, consistent with a role for ATM and XLF in stabilizing cleaved DNA ends and thus suppressing HJs (Bredemeyer et al., 2006, Lescale et al., 2016). In contrast, analysis of *IgkV*_*6-23*_*-J*_*1*_ rearrangements in PAXX-deficient pro-B cells did not reveal HJs, suggesting that PAXX, unlike its paralog XLF, most likely has a very minor, if any, role in stabilizing DNA ends within post-cleavage complexes (Figure 2D, see also below and Discussion).

### Coding and Signal Joint Defects in *Paxx*^*−/−*^*Xlf*^*−/−*^ pro-B Cells

**Figure 3:**
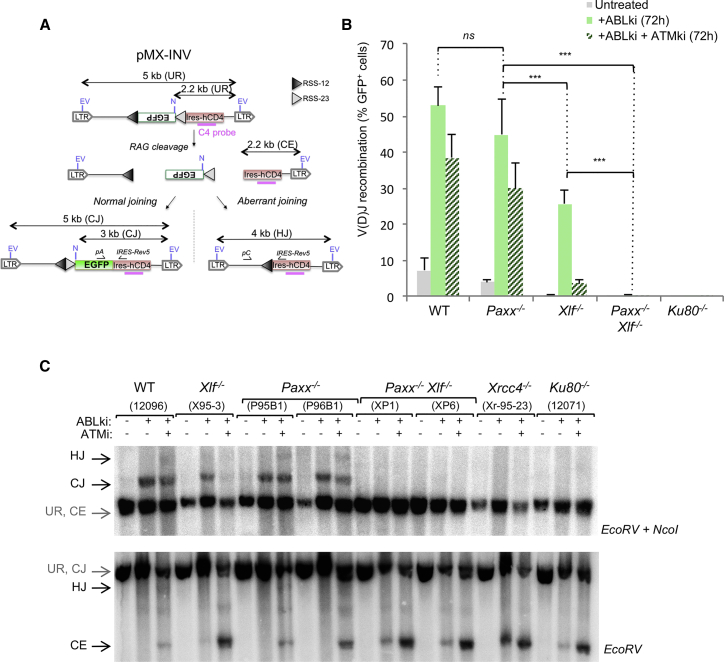
Defective Inversional V(D)J Recombination in *Paxx*^*−/−*^*Xlf*^*−/−*^ B Cells (A) Schematic of pMX-INV recombination substrate. The 12 recombination signal sequence (RSS-12; black triangle), GFP cDNA, 23 recombination signal sequence (RSS-23; gray triangle), internal ribosome entry site (IRES)-human CD4 cDNA (IRES-hCD4), long-terminal repeats (LTRs), EcoRV (EV) sites, NcoI (N) site, C4 probe (pink bar), and the expected sizes for the un-rearranged substrate (UR), coding end intermediates (CE), CJs and HJs are indicated. (B) *v-abl* pro–B cell lines treated for 72 hr with ABLki with or without ATM kinase inhibitor (ATMki) were assayed for pMX-INV rearrangement by flow cytometry with the percentage of GFP expressing cells indicated. Data represent mean ± SEM of at least four independent experiments using two WT (12095 and 12096), two *Paxx*^*−/−*^ (P96B1 and P96B13), four *Xlf*^*−/−*^ (16218, 16488, X95-3, and X95-4), two *Paxx*^*−/−*^*Xlf*^*−/*−^ (XP1 and XP6), and one *Ku80*^*−/−*^ (12071) independent cell lines. ^∗^, 0.01 ≤ p < 0.05; ^∗∗^, 0.001 ≤ p < 0.01; ^∗∗∗^, p < 0.001. (C) The indicated *v-abl* pro-B cell lines containing the pMX-INV substrate were treated for 72 hr with ABLki with or without ATMki and assayed by Southern blotting with EcoRV/NcoI digest-C4 probe (top) and EcoRV digest-C4 probe (bottom). See also Figure S2.

To unequivocally test for V(D)J recombination defects in PAXX/XLF-deficient cells, we transduced *v-abl* pro-B cell lines from each genotype with the pMX-RSS-GFP/IRES-hCD4 retroviral recombination substrate (pMX-INV) in which GFP is expressed upon successful chromosomal inversional RAG-mediated recombination and allows for the assessment of the rearrangement status and recombination intermediates by Southern blot analysis (Figure 3A) (Bredemeyer et al., 2006, Lescale et al., 2016). Both assays confirmed robust levels of rearrangements in ABLki-treated WT, *Xlf*^*−/*^,^*−*^ and *Paxx*^*−/−*^ cells (Figures 3B and 3C). Interestingly, although both *Paxx*^*−/−*^ and *Xlf*^*−/−*^ pro-B cells are proficient at recombining, we consistently found significantly higher levels of rearrangements in *Paxx*^*−/−*^ pro-B cells than *Xlf*^*−/−*^ pro-B cells (Figure 3B, 45% recombination in *Paxx*^*−/−*^ cells versus 26% recombination in *Xlf*^*−/−*^ cells, p < 0.001, Figure 3C). These results are reminiscent of the stronger radiation sensitivity observed in XLF-deficient cells in comparison to PAXX-deficient cells (Figure 1D) and suggest that XLF deficiency leads to a more profound defect in repairing DSB than PAXX deficiency.

Strikingly, flow cytometry analysis revealed severely impaired inversional rearrangement in *Paxx*^*−/−*^ *Xlf*^*−/−*^ cells in comparison to WT (≈115-fold decrease, p < 0.001), *Paxx*^*−/−*^ (≈97-fold decrease, p < 0.001), and *Xlf*^*−/−*^ (≈56-fold decrease, p < 0.001) cells (Figure 3B). Southern blot and PCR analysis confirmed severe end-joining defects in *Paxx*^*−/−*^ *Xlf*^*−/−*^ cells, as revealed by a marked decrease in CJs and a marked increase in unjoined CEs, indicative of a classical NHEJ defect (Figures 3C and S2). Notably, the intensity of the inversional V(D)J recombination defect in *Paxx*^*−/−*^ *Xlf*^*−/−*^ cells was similar to that of Ku80- and XRCC4-deficient cells (Figures 3B and 3C). *Paxx*^*−/−*^ *Xlf*^*−/−*^ cells treated with ABLki and the ATM-specific inhibitor Ku55933 (ATMki) (Hickson et al., 2004) showed stronger levels of unrepaired CEs in comparison to ABLki-treated *Paxx*^*−/−*^ *Xlf*^*−/−*^ cells, indicating that a fraction of unrepaired CEs are subjected to ATM-dependent end degradation in PAXX/XLF-deficient cells similar to what has been previously reported in the context of Ku80, core RAG2/XLF, H2AX/XLF, and 53BP1/XLF deficiency (Helmink et al., 2011, Lescale and Deriano, 2016, Zha et al., 2011).

**Figure 4:**
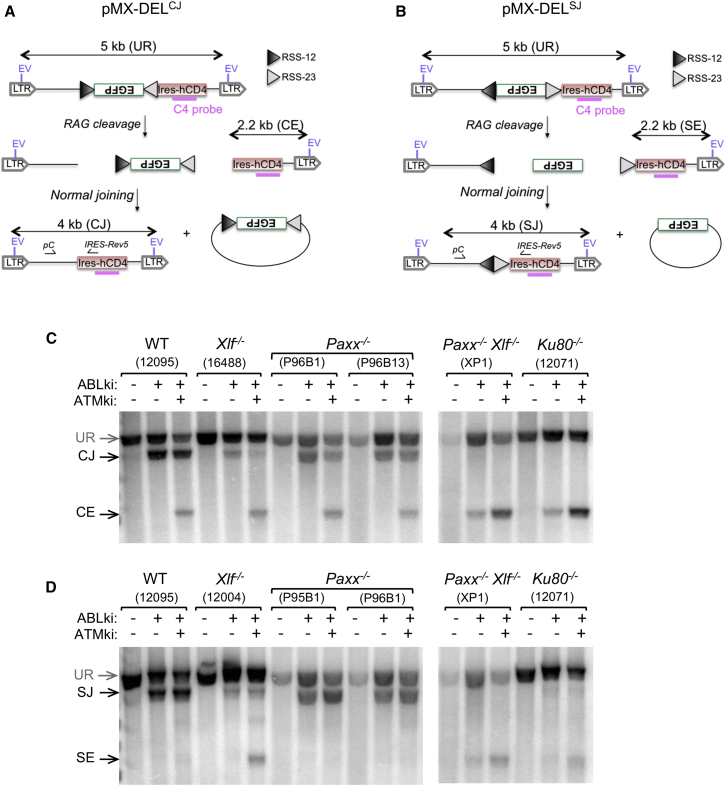
Defective Deletional V(D)J Recombination in *Paxx*^*−/−*^*Xlf*^*−/−*^ B Cells (A and B) Schematics of pMX-DEL^CJ^ (A) and pMX-DEL^SJ^ (B) recombination substrates with intermediates and products as defined for pMX-INV (Figure 3A). (C and D) *v-abl* pro-B cell lines containing pMX-DEL^CJ^ (C) or pMX-DEL^SJ^ (D) were treated for 72 hr with ABLki with or without ATMki and assayed by Southern blotting with EcoRV digest-C4 probe. See also Figures S3 and S4 and Table S4.

To identify specific defects in CJ and SJ formation, we generated multiple *v-abl* pro-B cell lines from each genotype that harbored either a chromosomal deletional substrate designed to assay CJs and unjoined CEs (pMX-DEL^CJ^; Figure 4A) or a chromosomal deletional substrate designed to assay SJs and unjoined SEs (pMX-DEL^SJ^; Figure 4B) (Bredemeyer et al., 2006). In agreement with the aforementioned observations, induction of RAG in WT, *Xlf*^*−/−*^, and *Paxx*^*−/−*^ cells generated substantial CJ and SJ levels with no obvious free CEs and SEs (Figures 4C, 4D, and S3). By contrast, *Paxx*^*−/−*^ *Xlf*^*−/−*^ cells had very little accumulation of CJs or SJs, which were only detectable after PCR amplification, and, instead accumulated un-joined CEs and SEs (Figures 4C and 4D and S3). To determine the fidelity of rare SJ formation in *Paxx*^*−/−*^ *Xlf*^*−/−*^ cells, we subjected SJ PCR products to digestion with the restriction enzyme *Apa*LI, which exclusively digests SJs formed without the loss or addition of nucleotides (Bogue et al., 1998, Ramsden et al., 1997). Unlike SJs formed in WT and PAXX-deficient pro-B cells, which are largely *Apa*LI sensitive, SJs derived from PAXX/XLF- and Ku80-deficient pro-B cells were almost exclusively resistant to *Apa*LI digestion, indicating that the rare SJs formed in these cells were imperfect (Figure S4A). SJs from XLF-deficient pro-B cells were also imperfect, although to a lesser extent than those observed in *Paxx*^*−/−*^ *Xlf*^*−/−*^ and *Ku80*^*−/−*^ cells (Figure S4). Consistently, sequencing of SJs derived from *Paxx*^*−/−*^ *Xlf*^*−/−*^ and *Ku80*^*−/−*^ cells revealed increased deletions and utilization of micro-homology in comparison to WT, *Paxx*^*−/−*^, and *Xlf*^*−/−*^ cells (Figures S4B–S4D; Table S4), demonstrating aberrant joining of signal ends by alternative NHEJ (Deriano and Roth, 2013) in PAXX/XLF-deficient pro-B cells. Taken together, these results provide strong evidence that PAXX and XLF double deficiency leads to a severe NHEJ defect in recombining lymphocytes.

### PAXX Function in V(D)J Recombination Does Not Overlap with ATM

Previous studies have shown that XLF functions in a cooperative manner with several members of the ATM-dependent DSB response pathway in joining DNA breaks during V(D)J recombination (Kumar et al., 2014, Zha et al., 2011). Because the ATM-DSB response relies on the kinase activity of ATM, treatment of XLF-deficient pro-B cells with an ATM-specific inhibitor abrogates V(D)J recombination in these cells (Kumar et al., 2014, Lescale et al., 2016, Zha et al., 2011). Based on the structural similarities that exist between XLF and PAXX (Ochi et al., 2015, Xing et al., 2015), we envisioned that PAXX might also be functionally redundant with the ATM-DSB response during V(D)J recombination. To test this hypothesis, we treated pMX-INV-transduced *v-abl* pro-B cell lines with ABLki and ATMki (Figures 3 and S2). As expected (Lescale et al., 2016, Zha et al., 2011), rearrangement was almost completely abolished in *Xlf*^*−/−*^ pro-B cells treated with ATMki, leading to a strong decrease in the percentage of GFP-positive pro-B cells (Figure 3B) and the accumulation of CEs instead of CJ products (Figure 3C). Inhibition of the ATM kinase activity in WT led to robust recombination (Figure 3B) and a specific accumulation of CEs (Figures 3C, 4C, 4D, S2, and S3) due to the role of ATM in stabilizing coding ends within post-cleavage complexes (Bredemeyer et al., 2006, Helmink and Sleckman, 2012). Strikingly, and in sharp contrast to XLF deficiency, pMX-INV rearrangement was not dramatically affected in *Paxx*^*−/−*^ pro-B cells treated with ATMki in comparison to untreated *Paxx*^*−/−*^ pro-B cells and ATMki-treated WT pro-B cells (Figures 3B, 3C, and S2). Consistently, we also observed robust CJ and SJ formation in pMX-DEL^CJ^ *Paxx*^*−/−*^ pro-B cells and pMX-DEL^SJ^ *Paxx*^*−/−*^ pro-B cells treated with ATMki, respectively (Figures 4C, 4D, and S3). Altogether, these results indicate that PAXX does not rely on ATM kinase activity during V(D)J recombination.

**Figure 5:**
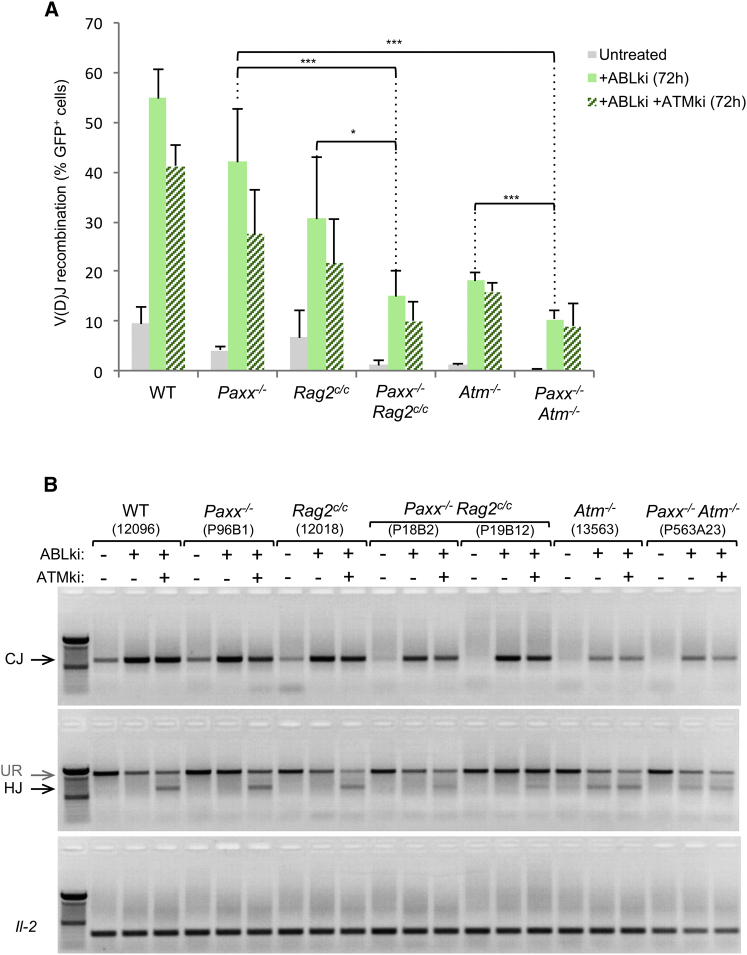
*Paxx*^*−/−*^*Atm*^*−/−*^ and *Paxx*^*−/−*^*Rag2*^*c/c*^ B Cells Perform Robust V(D)J Recombination (A) *v-abl* pro–B cell lines treated for 72 hr with ABLki with or without ATM kinase inhibitor (ATMki) were assayed for pMX-INV rearrangement by flow cytometry with the percentage of GFP expressing cells indicated. Data represent mean ± SEM of at least four independent experiments with two WT (12095 and 12096), two *Paxx*^*−/−*^ (P96B1 and P96B13), two *Rag2*^*c/c*^ (12018 and 12019), two *Paxx*^*−/−*^*Rag2*^*c/c*^ (P18B2 and P19B12), two *Atm*^*−/−*^ (160 and 13563), and one *Paxx*^*−/−*^*Atm*^*−/−*^ (P563A23) independent cell lines. ^∗^, 0.01 ≤ p < 0.05; ^∗∗^, 0.001 ≤ p < 0.01; ^∗∗∗^, p < 0.001. (B) PCR analysis of pMX-INV CJs and HJs from indicated *v-abl* abl pro-B cell lines treated for 72 hr with ABLki with or without ATMki. *Il-2* gene PCR was used as a loading control.

To further substantiate that PAXX function in V(D)J recombination is not redundant with ATM, we also generated PAXX/ATM doubly deficient pro-B cell lines by deleting *Paxx* from *Atm*^*−/−*^ pro-B cells (Figure S1; Table S1; data not shown). To quantify V(D)J recombination efficiency, we chromosomally integrated the pMX-INV substrate in pro-B cell clones (Figure 3A) and triggered RAG expression. In contrast to *Xlf*^*−/−*^ *Atm*^*−/−*^ pro-B cells (Zha et al., 2011) and *Paxx*^*−/−*^ *Xlf*^*−/−*^ pro-B cells (Figure 3B) and in agreement with our ATMki results (Figures 3 and 4), PAXX/ATM double knockout pro-B cells performed quite robust inversional recombination in comparison to ATM single knockout pro-B cells (Figures 5A and 5B). These results indicate that, although XLF and PAXX paralogs functionally overlap in repairing RAG-induced signal ends and coding ends, they diverge with regard to their respective redundancy with ATM.

### PAXX Function in V(D)J Recombination Does Not Overlap with the RAG2 C Terminus

We recently showed that XLF also cooperates with the RAG complex in repairing DNA breaks. Thus, in the context of RAG2 lacking the C terminus domain (*Rag2*^*c/c*^ mutant, also referred to as core RAG2), XLF deficiency leads to a profound lymphopenia associated with a severe defect in V(D)J recombination (Lescale et al., 2016). These results led us to propose a model in which the RAG proteins and the ATM-DSB response might participate in a same pathway (i.e., allowing the stabilization of cleaved DNA ends) that is functionally redundant with XLF (Lescale et al., 2016). To test for potential functional interaction between the RAG complex and PAXX, we deleted *Paxx* from core RAG2-expressing *v-abl* pro-B cell lines (Lescale et al., 2016) (Figure S1; Table S1). Induction of RAG in pMX-INV transduced *Paxx*^*−/−*^ *Rag2*^*c/c*^ pro-B cells led to robust recombination in comparison to core RAG2-expressing pro-B cells as measured by flow cytometry and PCR analysis (Figures 5A and 5B). In addition, pMX-INV rearrangement was not dramatically affected in *Paxx*^*−/−*^ *Rag2*^*c/c*^ pro-B cells treated with the ATMki in comparison to untreated *Paxx*^*−/−*^ *Rag2*^*c/c*^ pro-B cells and ATMki-treated *Rag2*^*c/c*^ pro-B cells (Figures 5A and 5B). Together, these results demonstrate that, unlike XLF, PAXX does not functionally overlap with the RAG complex in the repair of RAG-generated DNA ends. These findings also indicate that, during V(D)J recombination, the redundancy between XLF and PAXX is mechanistically distinct from the previously reported redundancy between XLF and RAG/ATM-DDR (Kumar et al., 2014, Lescale et al., 2016, Zha et al., 2011).

### PAXX Function in the Absence of XLF Depends on Its Interaction with Ku

**Figure 6:**
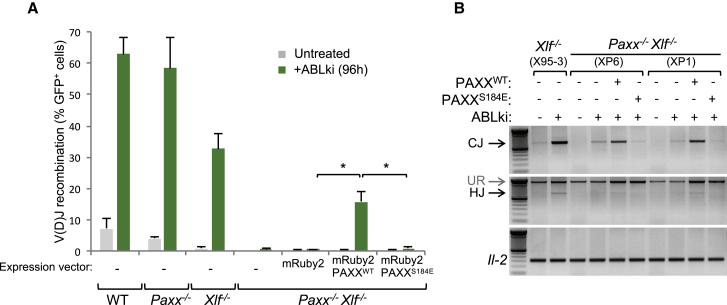
PAXX Function in the Absence of XLF Depends on Its Interaction with Ku (A) *Paxx*^*−/−*^*Xlf*^*−/*−^*v-abl* pro–B cell lines (XP1, XP6) were transfected with expression vectors encoding for mRuby2, mRuby2-PAXX^WT^, or mRuby2-PAXX^S184A^. After 6 hr recovery, *Paxx*^*−/−*^*Xl*^*−/*−^ transfected cells, along with controls, were treated for 96 hr with ABLki and assayed for pMX-INV rearrangement by flow cytometry with the percentage of GFP-expressing cells indicated. Data represent mean ± SEM of three independent experiments using two independent cell lines for each genotype. See also Figure S6. (B) PCR analysis of pMX-INV CJs and HJs from control and ABLki*-*treated *Xlf*^*−/−*^ and *Paxx*^*−/−*^*Xlf*^*−/*−^ pro-B cell lines expressing mRuby2-PAXX^WT^ or mRuby2-PAXX^S184A^. *Il-2* gene PCR was used as a loading control.

We recently reported that PAXX function during the repair of ionizing radiation-induced DNA damage depends on its ability to bind Ku (Ochi et al., 2015). PAXX-Ku interaction stimulates DNA end ligation by Ligase 4, promotes assembly of core NHEJ factors on damaged chromatin, and depends on the C terminus of PAXX, specifically residues V199 and F201 (Ochi et al., 2015). Here, we found that mutating the highly conserved residue S184 to glutamate (PAXX^S184E^) similarly abolishes PAXX interaction with Ku (Figure S5). To investigate whether the PAXX-Ku interaction is required for V(D)J recombination in the context of XLF deficiency, we expressed mRuby2 fluorescent protein (mRuby2), mRuby2-tagged WT PAXX protein (mRuby2-PAXX^WT^), and mRuby2-PAXX^S184E^ protein in pMX-INV *Paxx*^*−/−*^ *Xlf*^*−/−*^ pro-B cells (Figure 6). Expression of mRuby2-PAXX^WT^, but not mRuby2 and mRuby2-PAXX^S184E^, substantially restored recombination in RAG-induced *Paxx*^*−/−*^ *Xlf*^*−/−*^ pro-B cells (Figure 6A). In line with this, complementation with PAXX^WT^ in *Paxx*^*−/−*^ *Xlf*^*−/−*^, pro-B cells also led to the formation of CJs and HJs characteristic of XLF single deficiency (Lescale et al., 2016) (Figure 6B). To strengthen these results, we transfected *Paxx*^*−/−*^ *Xlf*^*−/−*^ pro-B cells carrying a modified m-Cherry version of the pMX-INV recombination substrate (Figure S6A) with expression vectors (Ochi et al., 2015) encoding for GFP, GFP-tagged PAXX^WT^, GFP-tagged C-terminally truncated PAXX^1–145^, and GFP-tagged PAXX^V199A/F201A^. Ectopic expression of GFP-PAXX^WT^, but not the Ku interaction-deficient proteins GFP-PAXX^1–145^ and GFP-PAXX^V199A/F201A^, partially restored RAG-mediated rearrangements in *Paxx*^*−/−*^ *Xlf*^*−/−*^ pro-B cells (Figure S6B). Together, these data support a model in which the PAXX-Ku interaction is crucial for PAXX function in V(D)J recombination.

## Discussion

**Figure 7:**
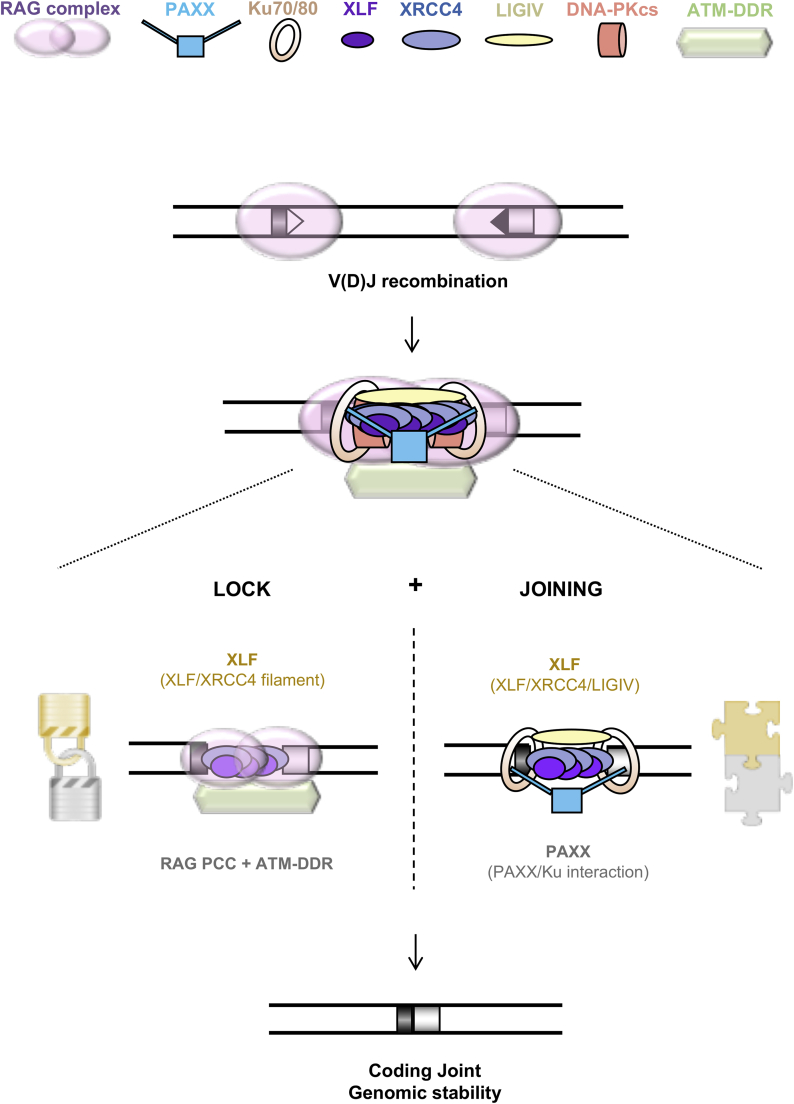
A Lock-and-Join Model for Repair of DNA Ends During V(D)J Recombination During V(D)J recombination, coding ends stay associated with the RAG proteins in a post-cleavage complex (RAG-PCC). Together with the ATM-dependent DNA damage response (ATM-DDR), the RAG-PCC contributes to stabilization (locking) of broken DNA ends. In the absence of RAG or ATM-DDR, XLF, most likely through the formation of XLF-XRCC4 hetero-filaments ensure tethering of DNA ends and the formation of CJs. Unlike its paralog XLF, PAXX does not seem to play major roles in locking broken coding ends. Instead, PAXX contributes to their joining, possibly by promoting the stimulation of the XRCC4/Ligase 4 enzymatic complex. This end-joining function is redundant with XLF and requires interaction with Ku.

PAXX, XRCC4, and XLF comprise a homologous superfamily of structurally related proteins that participate in the repair of DSBs by NHEJ (Ochi et al., 2015, Ochi et al., 2014). Here, we show that PAXX has a key role in V(D)J recombination that is masked by functional redundancy with XLF. Our results support a model in which the PAXX, XRCC4, and XLF paralogs play distinct redundant and nonredundant functions in the repair of RAG-generated DNA ends, specifically in the locking and joining steps, during antigen receptor gene assembly (Figure 7).

Recent studies have shown that XLF is redundant with several members of the ATM-DDR and the RAG complex in joining broken DNA ends (Deriano and Roth, 2013, Helmink and Sleckman, 2012, Kumar et al., 2014, Lescale et al., 2016, Zha et al., 2011). This redundancy is thought to rely, at least partially, on the ability of these complexes to assemble a synaptic complex that bridges DNA ends: the ATM-DDR through the formation of chromatin-associated DNA repair foci, the RAG complex through an unknown mechanism, and XLF via the formation of XLF/XRCC4 DNA end-bridging filaments. In contrast to XLF, we conclude that, based on several observations, PAXX does not participate in stabilizing RAG-DNA breaks (Figure 7). We reveal that *Paxx*^*−/−*^ *Atm*^*−/−*^ pro-B cells support robust V(D)J recombination, demonstrating that, unlike XLF, PAXX does not functionally overlap with ATM in repairing coding and signal ends (Figures 3–5, S2, and S3). Notably, we observed a small but significant decrease in overall recombination in *Paxx*^*−/−*^ *Atm*^*−/−*^ pro-B cells in comparison to *Paxx*^*−/−*^ pro-B cells and *Atm*^*−/−*^ pro-B cells (Figure 5A). This decrease could be attributed to additive NHEJ defects in the absence of PAXX and ATM (Craxton et al., 2015, Kumar et al., 2014, Ochi et al., 2015, Xing et al., 2015). We also found that, similar to PAXX/ATM deficiency, PAXX-deficient pro-B cells expressing the core RAG2 mutant protein (*Paxx*^*−/−*^ *Rag2*^*c/c*^) perform robust V(D)J recombination. Additionally, treatment of *Paxx*^*−/−*^ *Rag2*^*c/c*^ pro-B cells with a specific ATM kinase inhibitor does not abrogate recombination in these cells (Figure 5), indicating that, in the absence of a functional RAG/ATM-DDR complex, PAXX is not required for synapsis and joining of DNA ends. In line with this, destabilization of the post-cleavage synaptic complex in *Atm*^*−/−*^ and *Xlf*^*−/−*^ pro-B cells irremediably leads to the formation of HJs (Bredemeyer et al., 2006, Lescale et al., 2016); however, we did not detect HJs in recombining PAXX-deficient pro-B cells (Figure 2). Lastly, in line with previous results (Bredemeyer et al., 2006), we show that ATM deficiency leads to the accumulation of coding ends but not signal ends (Figure 4). This discrepancy is thought to rely on the proclivity of the RAG to bind more avidly to signal end pairs in comparison to coding end pairs (Agrawal and Schatz, 1997, Bredemeyer et al., 2006, Helmink and Sleckman, 2012, Hiom and Gellert, 1998, Schatz and Swanson, 2011). Combined ATM/XLF deficiency leads to an almost complete lack of CJs associated with an accentuated accumulation of coding ends and to the accumulation of signal ends, which is consistent with a redundant role for XLF in stabilizing DNA ends (Figure 4) (Zha et al., 2011). In sharp contrast, we find that PAXX/ATM deficiency does not affect coding and signal DSB intermediates beyond that of ATM single deficiency (Figures 4, 5, and S3). Altogether, our results strongly indicate that PAXX does not play a major role in stabilizing and tethering DNA ends during RAG-mediated rearrangements (Figure 7). Interestingly, Roy et al., (2015) have also recently shown that PAXX does not enhance the cellular requirement for XRCC4/XLF interaction and potential DNA end bridging in human epithelial cells.

We find that, upon RAG cleavage, more than 80% of *Paxx*^*−/−*^ *Xlf*^*−/−*^ pro-B cells accumulate 53BP1 DNA damage foci (Figures 2A and 2B). This is consistent with the accumulation of unrepaired RAG-DNA ends in these cells and in the context of other end-joining deficiency such as in XRCC4-, Ku80-, and core-RAG2/XLF-deficient cells (Figures 2–4, S2, and S3) (Lescale et al., 2016). Notably, a fraction of these 53BP1 foci might also arise due to under-replicated DNA (Harrigan et al., 2011). This possibility is in agreement with the observation that fibroblasts derived from immune-deficient patients carrying XLF mutations exhibit impaired cellular response to replication stress (Schwartz et al., 2009). It will be interesting to investigate the specific roles of XRCC4 paralogs and, more generally, components of the NHEJ pathway, during replication and to determine to which extent it contributes to the formation of 53BP1 foci in G1-arrested cells. Nonetheless, the complete lack of rearrangement and accumulation of unrepaired DNA ends observed in XLF/PAXX-deficient pro-B cells is consistent with a severe end-joining defect in these cells (Figures 2–4, S2, and S3). In line with a role for PAXX in NHEJ-mediated repair, we find that PAXX-Ku interaction is required for PAXX function in V(D)J recombination in the absence of XLF (Figures 6 and S6). Additionally, sequencing of rare joints in *Paxx*^*−/−*^ *Xlf*^*−/−*^ pro-B cells revealed increased end resection and micro-homology usage reminiscent of joints found in the absence of core NHEJ factors (Figure S4) (Deriano and Roth, 2013). Therefore, PAXX promotes the joining of coding and signal ends during V(D)J recombination (Figure 7).

The observation that *Xlf*^*−/−*^ cells are more radiosensitive than *Paxx*^*−/−*^ cells (Figure 1D) is consistent with our findings that PAXX and XLF have redundant (i.e., end-joining) and non-redundant (i.e., end-stabilization) functions in the context of the repair of RAG-generated DNA breaks. Thus, upon irradiation-induced DSBs, XLF-deficient cells might suffer from defects in both end-stabilization and end-joining whereas PAXX-deficient cells only display defects in end-joining. The more severe radiosensitivity observed in PAXX/XLF-deficient cells is congruent with a complete lack of DNA end joining in these cells. More surprising is our finding that PAXX/XLF-deficient pro-B cells have increased sensitivity to radiation-induced DNA damage than XRCC4-deficient pro-B cells. This result is reminiscent of the observation that XRCC4/XLF-deficient cells are more sensitive to hydroxyurea and zeocin than XRCC4-deficient cells (Roy et al., 2015). Considering that XLF function in stabilizing and joining DNA ends depends on XRCC4, these data indicate that XLF also plays XRCC4-independent function(s) during DSB repair.

The question remains of how PAXX rejoins RAG-cleaved DNA ends and whether this function is regulated. In response to genotoxic stress, ATM phosphorylates hundreds of proteins active in different aspects of the DNA damage response (Matsuoka et al., 2007). It will be interesting to test whether ATM regulates, possibly through phosphorylation, PAXX in response to RAG cleavage. This would potentially explain the severe end-joining defect observed in ATM/XLF cells (Kumar et al., 2014, Zha et al., 2011). XLF has been proposed to stimulate DSB ligation by promoting the adenylation of XRCC4/Ligase 4 (Riballo et al., 2009), and, in the absence of XLF, PAXX could potentially act as substitute for XLF in stimulating Ligase 4. Indeed, in vitro, both PAXX and XLF have been reported to stimulate XRCC4/Ligase 4-mediated end joining at certain types of DNA ends (Ochi et al., 2015, Xing et al., 2015). More work is needed to parse whether PAXX participates in joining DNA ends by directly stimulating Ligase 4 catalysis, promoting the assembly of core NHEJ factors to chromatin (Ochi et al., 2015), and/or promoting a yet undescribed mechanism.

Genetic mouse models and human patients with hereditary defects in NHEJ factors suffer from a large variety of defects, including radiation sensitivity, immunodeficiency, developmental defects, and predisposition to cancer (Revy et al., 2005, Rooney et al., 2004). It will be interesting to see whether *Paxx* knockout and *Paxx/Xlf* double knockout animals harbor such developmental and immunological defects and to what extent.

## Experimental Procedures

### CRISPR/Cas9 Gene Knockout in pro-B Cells

Please refer to Table S1 for a complete list of *v-abl* pro-B cell lines used in this study. All animal experiments were performed in accordance with the guidelines of the institutional animal care committee of Institut Pasteur/CEEA Ile-de-France-Paris1 under the protocol no. 2012-0036.

#### Generation of Paxx^−/−^ pro-B Cell Lines

v-abl pro-B cell lines were cultured in RPMI/15%FBS/Pen-Strep as previously reported (Lescale et al., 2016). Small-guide RNAs (sgRNAs) were designed with CRISPR DESIGN online tool (Zhang lab) and cloned into a MLM3636 vector. sgRNA-P1: 5′-CAGCAGGGCGGTCTCGCCGC-3′, sgRNA-P2: 5′-ATGCAACCTAGAGAGGCGGC-3′, and sgRNA-P3: 5′-ACTAGAGGTTGAAGTCGTCG-3′. Two strategies were set up to inactivate *Paxx* gene. The first one deleted all of exons 1–4 (sgRNA-P1 + sgRNA-P2), removing the ATG start codon. The second strategy (sgRNA-P2 + sgRNA-P3) deleted part of exons 1–4, contributing to β sheets 3–7 and α helixes 1–3 (Xing et al., 2015) and led to frameshift and/or stop mutations 3′ of sgRNA-P2 in all clones used in this study (Figure 1 and Table S1). 15 million pro-B cells were transfected using the Cell Line Nucleofector Kit V from Lonza (program X-001, Amaxa Nucleofector Technology) and 10 μg of plasmid. Electroporated cells were resuspended in regular RPMI/FBS/Pen-Strep medium at a density of 1 × 10^6^ cells/ml. After 24–48 hr recovery, cells were isolated in 96-well plates by single-cell sorting with an ARIA II (BD Biosciences). After 1 week, clones were screened by PCR using primers 5′-ATGAGAGACTCCCCTGGACA 3′ and 5′-ACCCGGAAACAATGTCAACC-3′ amplifying around the expected deletion site. The absence of the protein was confirmed by western blot (see Figure 1).

#### Generation of Paxx^−/−^ Atm^−/−^ pro-B cell Lines

*Paxx*^*−/−*^ *Atm*^*−/−*^ pro-B cell clones were generated by deleting *Paxx* from established *Atm*^*−/−*^ pro-B cell lines as described previously.

#### Generation of Paxx^−/−^ Rag2^c/c^ pro-B Cell Lines

*Paxx*^*−/−*^ *Rag2*^*c/c*^ pro-B cell clones were generated by deleting *Paxx* from established *Rag2*^*c/c*^ pro-B cell lines as described previously.

#### *Generation of Xlf*^*−/−*^ and *Paxx*^*−/−*^*Xlf*^*−/−*^*pro-B Cell Lines*

*Xlf*^*−/−*^ and *Paxx*^*−/−*^ *Xlf*^*−/−*^ pro-B cell clones were generated by deleting *Xlf* exon 1 from WT and *Paxx*^*−/−*^ pro-B cells, respectively, using sgRNA-X1: 5′-TTAGCATACACCAACTTC-3′ and sgRNA-X2: 5′-CACCAACAGGTACTCATA-3′. Clones were screened by PCR using primers 5′-ACAAGGTCTAATGCACCCCA-3′ and 5′-GGGTTGCAGCCTTAGAAAAGT-3′.

#### Generation of Xrcc4^−/−^ pro-B Cell Lines

*Xrcc4*^*−/−*^ pro-B cell clones were generated by deleting part of *Xrcc4* exon 3 from WT pro-B cells using sgRNA-Xr1: 5′-GAATGTATAACAGGAGACGG-3′ and sgRNA-Xr2: 5′-GCCGAGACTCCTTAGAAAAG-3′. Clones were screened by PCR using primers 5′-CCCTCACAGAAACACAACTCA-3′ and 5′-CAAGGAGGTGGCCACTAGTT-3′.

### Irradiation Sensitivity Assay

Pro-B cells were plated in 12-well plates at 0.5 × 10^6^ cells/ml. Cells were irradiated at 0, 1, 2.5, or 5 Gy (Faxitron X-ray). 3 days after irradiation, viable cells were counted with a Casy cell counter (Roche). The percentages of viable cells compared to non-irradiated control cells were determined. Experiments were performed using two independent cell lines of each genotype and were repeated three times (see Table S2).

### Western Blotting

Cells were lysed using RIPA cell lysis reagent (Thermo Fisher Scientific) and protease inhibitors cocktail (Sigma-Aldrich). Equal amounts of proteins were subjected to SDS-PAGE on 4%–12% Bis-Tris gel. Proteins were transferred onto a nitrocellulose membrane (Life Technologies) using the iBlot apparatus (P3 program, 7 min transfer, Invitrogen). The membrane was exposed to Ponceau red staining (Sigma-Aldrich), washed, incubated in 5% non-fat dried milk in TBS containing 0.05% Tween-20 buffer for 1 hr at room temperature (RT), and subsequently incubated overnight at 4°C with primary antibody against PAXX protein (ab126353, 1:1000 dilution, Abcam) and γ-Tubulin protein (T6557, 1:20,000 dilution, Sigma-Aldrich). The membrane was then washed three times with TBS-Tween before incubation for 1 hr at RT with HRP-conjugated antibodies (7,074 or 7,076, 1:20,000 dilution, Cell Signaling Technology). Immune complexes were detected with WesternBright Sirius substrate (Advansta).

### V(D)J Recombination Assays

The pMX-INV, pMX-DEL^CJ^, or pMX-DEL^SJ^ substrates were introduced in pro-B cell lines through retroviral infection, and cells that had integrated the recombination substrate were enriched based on hCD4 expression (Bredemeyer et al., 2006, Liang et al., 2002). For V(D)J recombination assay, *v-abl*-transformed, *Bcl2*/pMX-INV-infected pro-B cells (10^6^/ml) were treated with 3 μM of the *v-abl* kinase inhibitor STI571 (referred to as ABLki in this study, Novartis) and assayed for rearrangement by FACS analysis of GFP expression or Southern blotting at 0, 72, and/or 96 hr. In some experiments, the ATM kinase inhibitor KU55933 was added at 15 μM together with STI571. For FACS analysis, V(D)J recombination efficiency was scored as the percentage of GFP-positive cells among hCD4-positive cells (human CD4-PE, Miltenyi Biotec, 1:20 dilution). The pMX-INV-mCherry substrate was built by replacing the inverted GFP cDNA from pMX-RSS-GFP/IRES-hCD4 (pMX-INV) by an inverted mCherry cDNA.

### Southern Blot

Southern blots were performed as previously described (Lescale et al., 2016). 50 μg of gDNA from untreated, ABLki-treated and ABLki/ATMki-treated pro-B cell lines were digested overnight with EcoRV for both pMX-DEL^CJ^ and pMX-DEL^SJ^ and with EcoRV or EcoRV/NcoI for pMX-INV. Digested gDNA samples were run overnight on an agarose gel, denatured by incubating the gel with 0.5 M NaOH/0.6M NaCl for 1 hr, and then transferred overnight on a Zeta-Probe GT nylon membrane (BioRad). DNA was cross-linked on the membrane using a UV Cross-linker CL-508 (Uvitec Cambridge). Blots were incubated at 42°C in pre-hybridization buffer for at least 1 hr and then overnight in hybridization buffer containing 32^P^-CTP-labeled C4 probe (Bredemeyer et al., 2006). Blots were washed in 2XSSC/0.1% SDS at 65°C and exposed to a Storage Phosphor Screen (GE Healthcare) for 2 to 5 days. The screen was then scanned using a Storm 860 PhosphoImager (Molecular Dynamics).

### PCR Analysis of V(D)J Recombination Products

pMX-DEL^CJ^ CJs, pMX-DEL^SJ^ SJs, and pMX-INV HJs were amplified using pC (GCACGAAGTCTTGAGACCT) and IRES-REV5 (CTCGACTAAACACATGTAAAGC) oligonucleotides. pMX-INV CJs were amplified using pA (CACAACATCGAGGACGG) and IRES-REV5 primers as previously described (Bredemeyer et al., 2006, Helmink et al., 2011). *Il-2* gene was amplified using IMR42 (CTAGGCCACAGAATTGAAAGATCT) and IMR43 (GTAGGTGGAAATTCTAGCATGATGC) primers and was used as loading control. pMX-DEL^SJ^ SJ PCR products were incubated with the restriction enzyme *Apa*LI (New England Biolabs) for 2 hr at 37°C. pMX-DEL^SJ^ SJs were cloned using TOPO TA Cloning kit (Life Technologies) following the manufacturer’s instructions and analyzed with Sanger Sequencing using T3 (AATTAACCCTCACTAAAGGGA) and T7 (TAATACGACTCACTATAGG) primers.

Endogenous *Vκ*_*6-23*_*/Jκ*_*1*_ coding and HJs were amplified as previously described (Bredemeyer et al., 2006). 500 ng of genomic DNA was amplified using pκJa (GGAGAGTGCCAGAATCTGGTTTCAG) and pκ6a (TGCATGTCAGAGGGCACAACTG) primers for HJ and pkJa2 (GCCACAGACATAGACAACGGAA) and pκ6d (GAAATACATCAGACCAGCATGG) primers for CJ. Serial 4-fold dilutions of this reaction were amplified using pκJa and pκ6b (CTACCAAACTTTGCAACACACAGGC) primers for HJ and pkJa2 and pκ6c (GTTGCTGTGGTTGTCTGGTG) primers for CJ.

### Immunofluorescence on Interphase Nuclei

After 3 days of treatment with ABLki, pro-B cell lines were adhered to poly-L lysine-coated coverslips and stained as previously described (Chaumeil et al., 2013, Lescale et al., 2016). Cells were fixed with 4% paraformaldehyde/PBS for 10 min at RT and permeabilized for 5 min with 0.4% Triton/PBS on ice. Immunofluorescence was performed after 30 min blocking in 2.5% BSA/10% goat serum/0.1% Tween-20/PBS, with a primary antibody against 53BP1 (NB100-304SS, 1:600 dilution, Novus Biologicals) and a secondary goat-anti-rabbit antibody (Alexa Fluor 594, 1:900 dilution; Life Technologies) in blocking solution for 1 hr each at RT. Cells were washed three times with 0.5% BSA/0.1% Tween-20/PBS after both primary and secondary antibody incubations. Finally, slides were mounted in ProLong Gold (Life Technologies) containing DAPI) to counterstain total DNA. Cells were imaged in 3D (nine z stacks of 0.5 μm) using a Zeiss AxioImager Z2 microscope and the Metacyte automated capture system (Metasystems). 53BP1 foci were counted using a custom Metacyte classifier. >10,000 nuclei were counted for each genotype (see Table S3).

### PAXX Expression Vectors

Plasmids encoding for GFP-tagged PAXX have been described previously (Ochi et al., 2015). DNA encoding mRuby2-tagged PAXX was synthesized and cloned into pcDNA3.1/Zeo(+) vector (Life Technologies). S184E mutation was introduced by site-directed mutagenesis (Agilent Technologies). Plasmids were transfected into 293FT cells using Lipofectamine 2000 (Life Technologies) for subsequent immunoprecipitation experiments.

### Immunoprecipitation

GFP pull-downs were carried out in 293FT cells exactly as previously described (Blackford et al., 2015). Antibody recognizing Ku80 (MS-285-P1, Thermo Scientific) was diluted 1:2,000 for western blotting experiments.

### PAXX Complementation of *v-abl* pro-B Cells

pMX-INV-GFP/mCherry pro-B cells were transfected with plasmids encoding for PAXX and mutated PAXX using the Cell Line Nucleofector Kit V from Lonza using the same conditions as for the previously described CRISPR/Cas9 gene knockout strategy. Cells were left to recover in fresh media for 6 hr and subsequently treated with ABLki and assayed for V(D)J recombination by FACS analysis as previously described.

### Statistics

All statistical analyses were performed using a non-parametric Mann-Whitney test. In all statistical tests, p < 0.05 were taken to be significant (^∗^, 0.01 ≤ p < 0.05; ^∗∗^, 0.001 ≤ p < 0.01; ^∗∗∗^, p < 0.001).

## Author Contributions

C.L. and H.L.H. performed the experiments. J.J.B. designed and generated sgRNA-expressing vectors. W.Y. generated *Xrcc4*^*−/−*^ pro-B cell clones. L.B. and A.J. contributed to experiments. C.C. generated the mCherry-pMX-INV substrate. R.S. generated the mRuby2-PAXX-S184E construct. B.R.-S.-M. provided the sgRNA-CRISPR/Cas9 vector targeting *Xlf*. A.N.B., G.B., and S.P.J. provided the PAXX-expressing vectors, performed PAXX/Ku immunoprecipitations, and commented on the manuscript. C.L., H.L.H., and L.D. designed the study, analyzed the data, and wrote the manuscript.

## Supplemental Information

Document S1. Figures S1–S6 and Tables S1–S4 Document S1. Figures S1–S6 and Tables S1–S4

Document S2. Article plus Supplemental Information Document S2. Article plus Supplemental Information
