# Supplementary material for: Specific Roles of XRCC4 Paralogs PAXX and XLF during V(D)J Recombination
Source: Cell Rep. 2016 Sep 2;16(11):2967–79. doi: 10.1016/j.celrep.2016.08.069 (PMC5033762; doi:10.1016/j.celrep.2016.08.069)
Supplement: Document S1. Figures S1–S6 and Tables S1–S4 [file mmc1.pdf]

**Cell Reports, Volume *16***

## Supplemental Information

# Specific Roles of XRCC4 Paralogs PAXX and XLF during V(D)J Recombination

**Chloé Lescale, Hélène Lenden Hasse, Andrew N. Blackford, Gabriel Balmus, Joy J. Bianchi, Wei Yu, Léa Bacoccina, Angélique Jarade, Christophe Clouin, Rohan Sivapalan, Bernardo Reina-San-Martin, Stephen P. Jackson, and Ludovic Deriano**

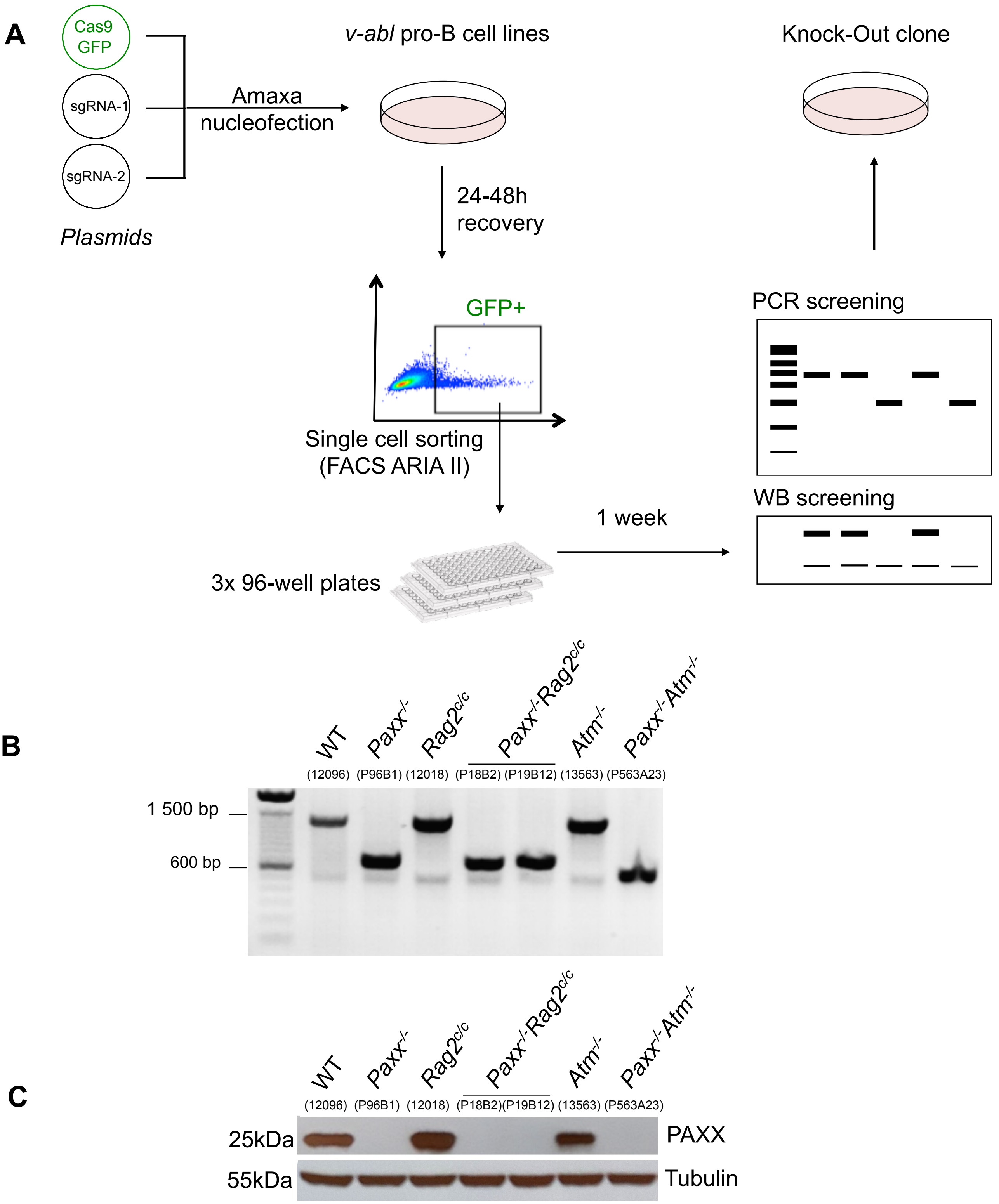


**Figure S1. Experimental strategy, Related to Figure 1**
(A) Scheme depicting the experimental strategy for CRISPR/Cas9 genome editing of pro-B cell lines.
(B) PCR analysis showing the deletion of approximately 626bp in the *Paxx* gene (WT = 1299bp band; *Paxx* KO ≈ 673bp band).
(C) Western blot showing the absence of the PAXX protein in *Paxx*$^{-/-}$ *Rag2*$^{c/c}$ and *Paxx*$^{-/-}$ *Atm*$^{-/-}$ pro-B cell clones.

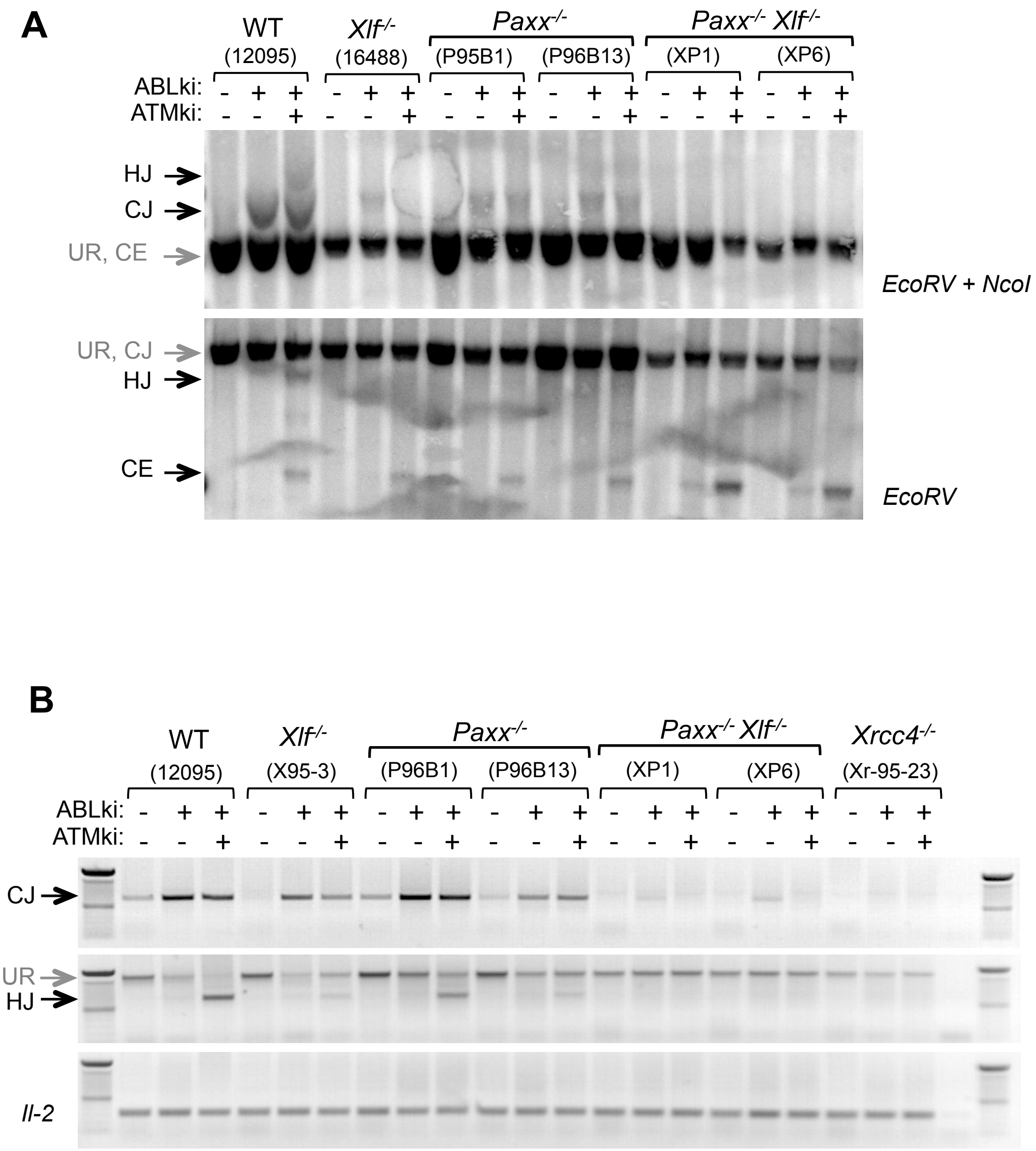


**Figure S2. Defective inversional V(D)J recombination in *Paxx*$^{-/-}$ *Xlf*$^{-/-}$ B cells, Related to Figure 3**
(A) The indicated *v-abl* pro-B cell lines containing the pMX-INV substrate were treated for 72h with ABLki with or without ATMki and assayed by Southern blotting; EcoRV/NcoI digest – C4 probe (top panel) and EcoRV digest – C4 probe (bottom panel).
(B) PCR analysis of pMX-INV coding and hybrid joints from indicated *v-abl* abl pro-B cell lines treated for 72h with ABLki with or without ATMki. *Il-2* gene PCR was used as a loading control.

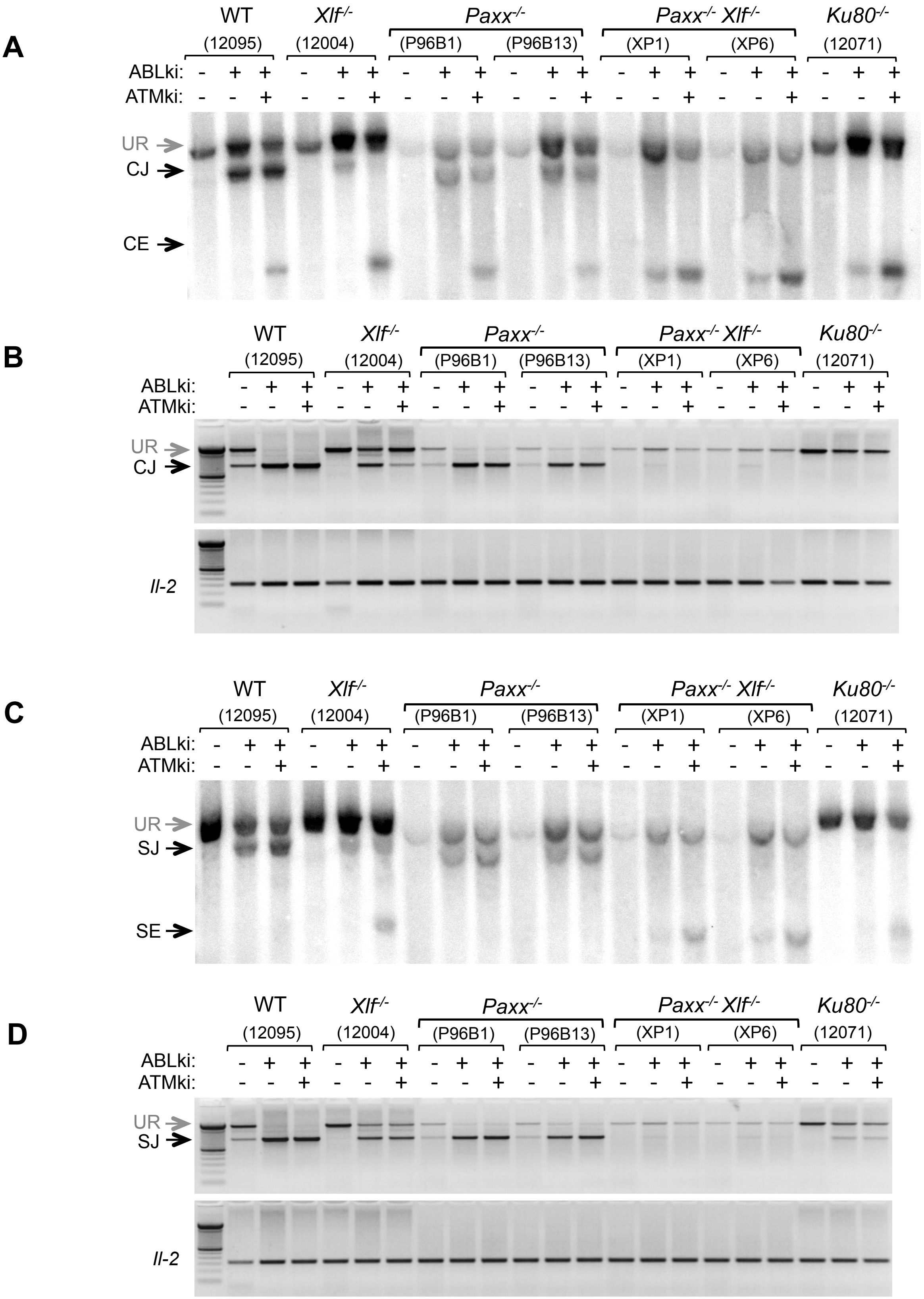


**Figure S3. Defective deletional coding and signal joint formation in *Paxx*$^{-/-}$ *Xlf*$^{-/-}$ B cells, Related to Figure 4**

pMX-DEL$^{CJ}$ (A, B) and pMX-DEL$^{SJ}$ (C, D) *v-abl* pro-B cell lines were treated for 72h with ABLki with or without ATMki. Coding and signal joint formation were assessed by Southern blotting; EcoRV digest – C4 probe (A, C) and by PCR (B, D). *Il-2* gene PCR was used as a loading control (B, D).

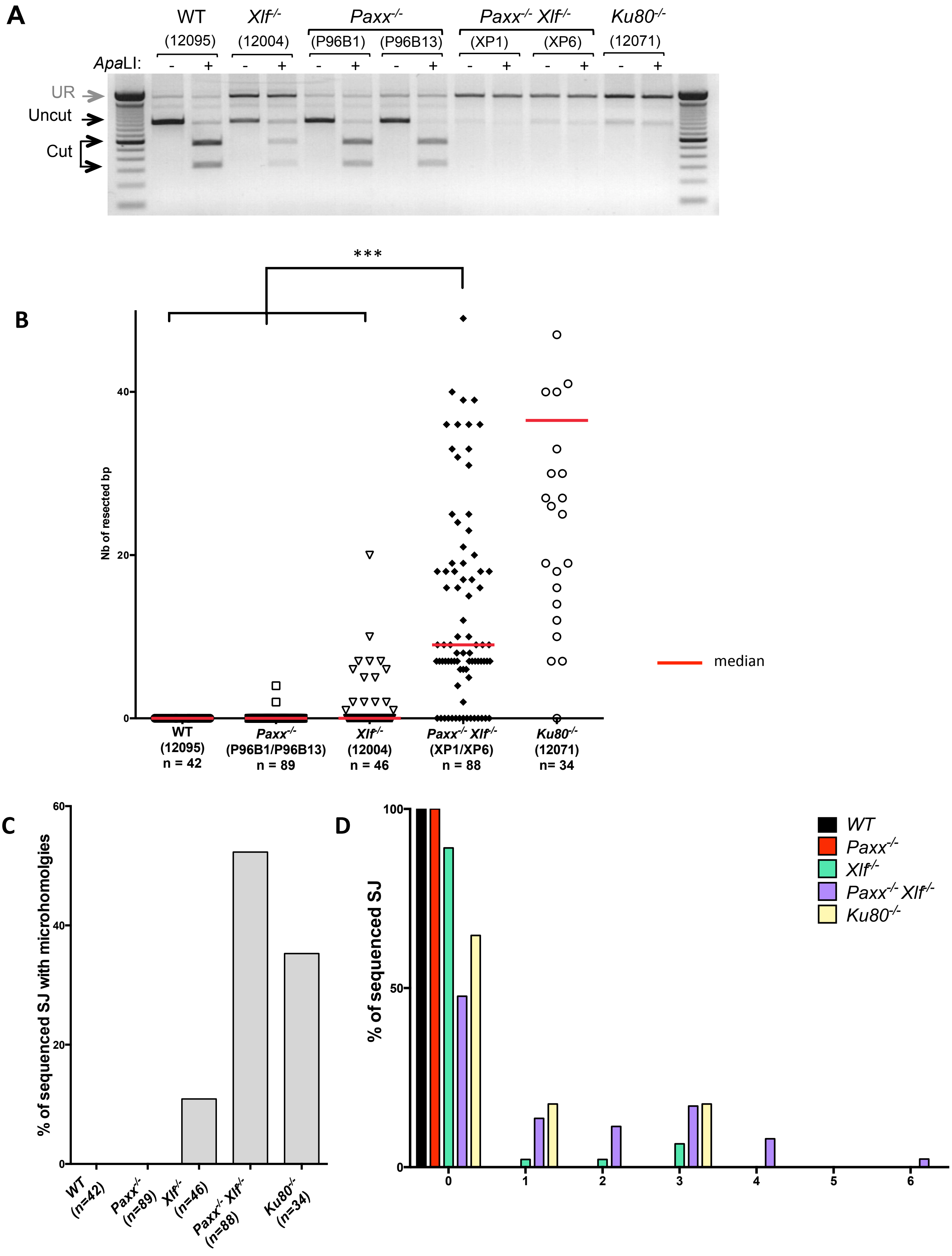
A
WT
(12095)
Xlf-/-
(12004)
Paxx-/-
(P96B1)
(P96B13)
Paxx-/- Xlf-/-
(XP1)
(XP6)
Ku80-/-
(12071)
ApaLI:
UR
Uncut
Cut
B
***
Nb of resected bp
WT (12095) n = 42
Paxx-/- (P96B1/P96B13) n = 89
Xlf-/- (12004) n = 46
Paxx-/- Xlf-/- (XP1/XP6) n = 88
Ku80-/- (12071) n= 34
median
C
% of sequenced SJ with microhomolgies
WT (n=42)
Paxx-/- (n=89)
Xlf-/- (n=46)
Paxx-/- Xlf-/- (n=88)
Ku80-/- (n=34)
D
% of sequenced SJ
WT
Paxx-/-
Xlf-/-
Paxx-/- Xlf-/-
Ku80-/-

**Figure S4. Analysis of signal joints sequences in *v-abl* pMX-DEL$^{SJ}$ pro-B cell lines, Related to Figure 4**

(A) PCR products of pMX-DEL$^{SJ}$ signal junctions were digested by *Apa*LI.

(B) Sanger sequencing of PCR amplified signal joints from *v-abl* pro-B cells (See also Table S4). Number of base-pair deletions is indicated on the y-axis. The horizontal bar represents the median. The total number of sequenced signal joints (n) is indicated. Outliers (> 49 bp deletion) are not shown but are included in calculation of the median. ***$p<0.001$.

(C and D) Microhomology utilization in pMX-DEL$^{SJ}$ SJs sequenced from indicated *v-abl* pro-B cell lines. (C) Percentage of sequences with microhomologies ≥ 1bp. (D) Microhomology length distribution among the same sequences.

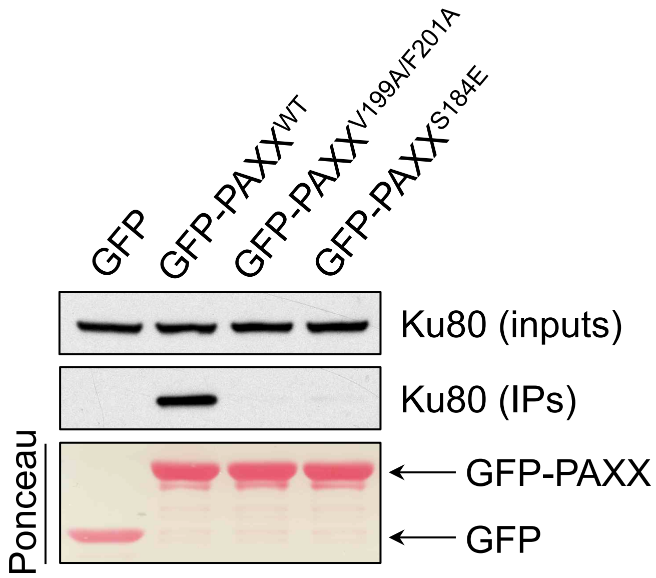


**Figure S5. C-terminal PAXX mutations S184E or V199A/F201A similarly abolish the PAXX-Ku interaction, Related to Figure 6**

Pulldowns assays from 293FT cells transiently transfected with the indicated plasmids.

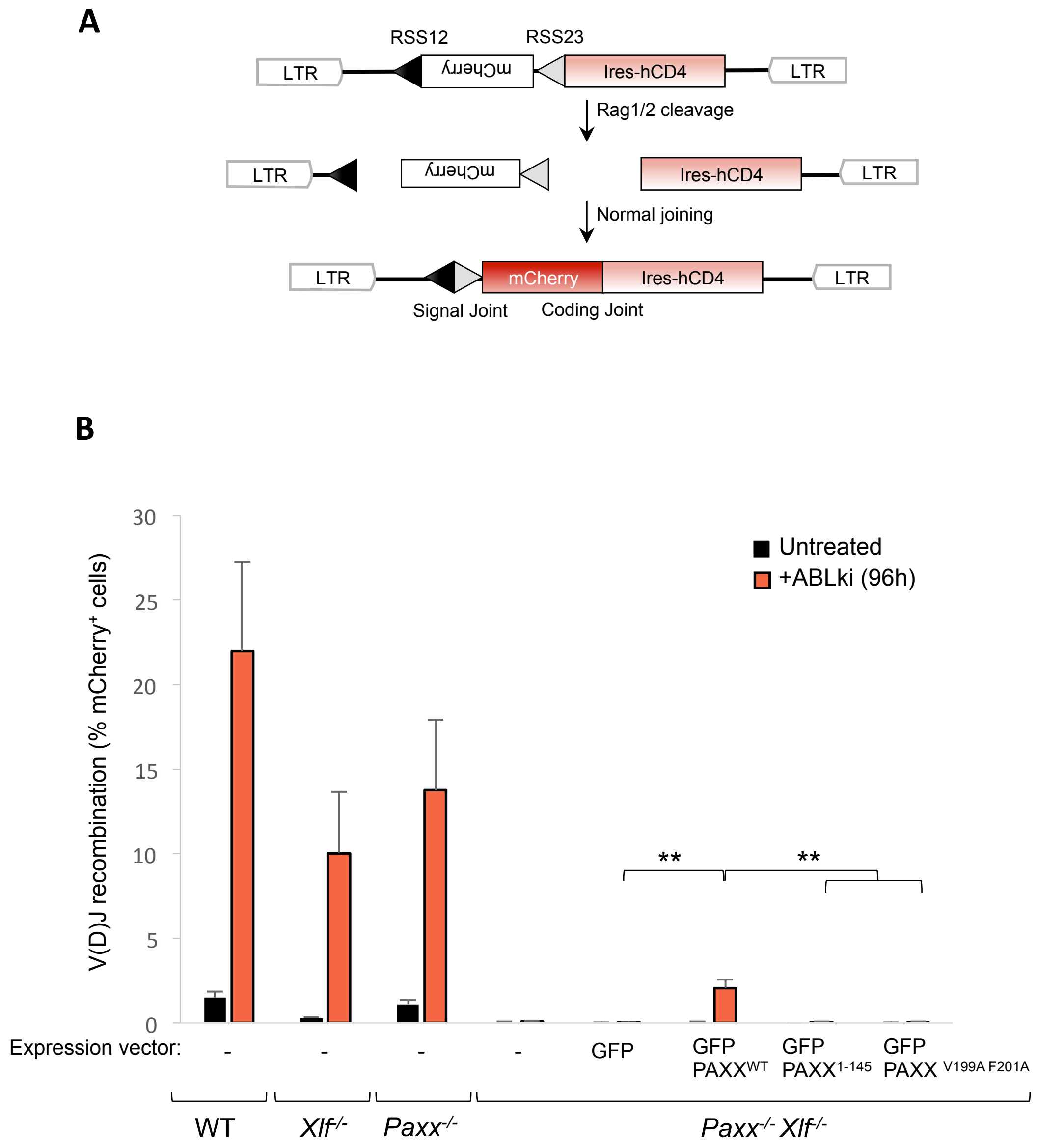


**Figure S6. PAXX function in the absence of XLF depends on its interaction with Ku, Related to Figure 6**

(A) Schematic representation of pMX-INV-Cherry recombination substrate.

(B) *Paxx*$^{-/-}$ *Xlf*$^{-/-}$ pro–B cell lines (XP1, XP6) were transfected with expression vectors encoding for GFP, GFP-PAXX$^{WT}$, GFP-PAXX$^{1\text{-}145}$ or GFP-PAXX$^{V199A/F201A}$. After 6h recovery, *Paxx*$^{-/-}$ *Xlf*$^{-/-}$ transfected cells, along with control untransfected cells, were treated for 96 hours with ABLki and assayed for pMX-INV-Cherry rearrangement by flow cytometry, with the percentage of mCherry expressing cells indicated. Data represent the means ± SEMs of three experiments with two independent cell lines for *Paxx*$^{-/-}$ *Xlf*$^{-/-}$ cell lines and one for the other cell lines.

| Genotype | Cell line # | Generation method | Reference |
|---|---|---|---|
| **WT** | 12096 | *v-Abl/Bcl2* immortalization of bone marrow pro-B cells | Lescale et al., 2016 |
| | 12095 | *v-Abl/Bcl2* immortalization of bone marrow pro-B cells | Lescale et al., 2016 |
| ***Paxx$^{-/-}$*** | P96B1 | CRISPR-Cas9 genome editing of 12096 cell line | This study |
| | P96B13 | CRISPR-Cas9 genome editing of 12096 cell line | This study |
| | P95B1 | CRISPR-Cas9 genome editing of 12095 cell line | This study |
| ***Paxx$^{-/-}$ Xlf$^{-/-}$*** | XP1 | CRISPR-Cas9 genome editing of P96B1 cell line | This study |
| | XP6 | CRISPR-Cas9 genome editing of P96B1 cell line | This study |
| ***Xlf$^{-/-}$*** | X95-3 | CRISPR-Cas9 genome editing of 12095 cell line | This study |
| | X95-4 | CRISPR-Cas9 genome editing of 12095 cell line | This study |
| | 16488 | *v-Abl/Bcl2* immortalization of bone marrow pro-B cells | This study |
| | 12004 | *v-Abl/Bcl2* immortalization of bone marrow pro-B cells | Lescale et al., 2016 |
| ***Ku80$^{-/-}$*** | 12071 | *v-Abl/Bcl2* immortalization of bone marrow pro-B cells | Lescale et al., 2016 |
| ***Xrcc4$^{-/-}$*** | Xr-95-21 | CRISPR-Cas9 genome editing of 12095 cell line | This study |
| | Xr-95-23 | CRISPR-Cas9 genome editing of 12095 cell line | This study |
| ***Rag2$^{c/c}$*** | 12018 | *v-Abl/Bcl2* immortalization of bone marrow pro-B cells | Lescale et al., 2016 |
| | 12019 | *v-Abl/Bcl2* immortalization of bone marrow pro-B cells | Lescale et al., 2016 |
| ***Paxx$^{-/-}$ Rag2$^{c/c}$*** | P18B2 | CRISPR-Cas9 genome editing of 12018 cell line | This study |
| | P19B12 | CRISPR-Cas9 genome editing of 12019 cell line | This study |
| ***Atm$^{-/-}$*** | 13563 | *v-Abl/Bcl2* immortalization of bone marrow pro-B cells | This study |
| | 160 | *v-Abl/Bcl2* immortalization of bone marrow pro-B cells | This study |
| ***Paxx$^{-/-}$ Atm$^{-/-}$*** | P563A23 | CRISPR-Cas9 genome editing of 13563 cell line | This study |

**Table S1. List and origin of *v-abl* pro-B cell lines, Related to Figure 1**

**A**

| EXPMT 1 | Cell line # | NI | 1 Gy | 2.5 Gy | 5 Gy |
|---|---|---|---|---|---|
| **WT** | 12095 | 4.3 (100%) | 4.12 (95.8%) | 3.42 (79.5%) | 1.49 (34.7%) |
| | 12096 | 4.4 (100%) | 4.31 (98.0%) | 3.43 (78.0%) | 1.11 (25.2%) |
| ***Paxx$^{-/-}$*** | P96B1 | 4.3 (100%) | 4.11 (95.6%) | 2.27 (52.8%) | 0.7 (16.3%) |
| | P96B13 | 4.5 (100%) | 4.0 (88.9%) | 2.78 (61.8%) | 0.79 (17.6%) |
| ***Xlf$^{-/-}$*** | 16488 | 3.6 (100%) | 2.17 (60.3%) | 0.84 (23.3%) | 0.37 (10.3%) |
| | X95-3 | 4.0 (100%) | 3.58 (89.5%) | 1.98 (49.5%) | 0.57 (14.3%) |
| ***Paxx$^{-/-}$ Xlf$^{-/-}$*** | XP1 | 4.4 (100%) | 1.8 (40.9%) | 0.69 (15.7%) | 0.38 (8.6%) |
| | XP6 | 4.7 (100%) | 1.97 (41.9%) | 0.76 (16.2%) | 0.41 (8.7%) |
| ***Xrcc4$^{-/-}$*** | Xr-95-21 | 3.8 (100%) | 2.9 (76.3%) | 0.81 (21.3%) | 0.35 (9.2%) |
| | Xr-95-23 | 4.2 (100%) | 3.79 (90.2%) | 1.68 (40.0%) | 0.51 (12.1%) |

| EXPMT 2 | Cell line # | NI | 1 Gy | 2.5 Gy | 5 Gy |
|---|---|---|---|---|---|
| **WT** | 12095 | 4.8 (100%) | 4.23 (88.1%) | 3.47 (72.3%) | 2.0 (41.7%) |
| | 12096 | 4.28 (100%) | 3.78 (88.3%) | 2.82 (65.9%) | 1.17 (27.3%) |
| ***Paxx$^{-/-}$*** | P96B1 | 4.43 (100%) | 3.4 (76.7%) | 2.78 (62.8%) | 0.87 (19.6%) |
| | P96B13 | 4.54 (100%) | 4.2 (92.5%) | 2.6 (57.3%) | 1.0 (22.0%) |
| ***Xlf$^{-/-}$*** | 16488 | 3.83 (100%) | 3.2 (83.6%) | 1.65 (43.1%) | 0.63 (16.4%) |
| | X95-3 | 4.55 (100%) | 3.48 (76.5%) | 2.06 (45.3%) | 0.72 (15.8%) |
| ***Paxx$^{-/-}$ Xlf$^{-/-}$*** | XP1 | 4.48 (100%) | 1.52 (33.9%) | 1.39 (31.0%) | 0.51 (11.4%) |
| | XP6 | 4.86 (100%) | 1.98 (40.7%) | 1.2 (24.7%) | 0.6 (12.3%) |
| ***Xrcc4$^{-/-}$*** | Xr-95-21 | 4.29 (100%) | 2.76 (64.3%) | 1.29 (30.1%) | 0.47 (11.0%) |
| | Xr-95-23 | 4.33 (100%) | 3.62 (83.6%) | 2.43 (56.1%) | 1.0 (23.1%) |

| EXPMT 3 | Cell line # | NI | 1 Gy | 2.5 Gy | 5 Gy |
|---|---|---|---|---|---|
| **WT** | 12095 | 4.28 (100%) | 4.3 (100.5%) | 3.7 (86.4%) | 2.4 (56.1%) |
| | 12096 | 4.32 (100%) | 3.77 (87.3%) | 3.43 (79.4%) | 2.11 (48.8%) |
| ***Paxx$^{-/-}$*** | P96B1 | 4.54 (100%) | 3.8 (83.7%) | 3.04 (67.0%) | 1.68 (37.0%) |
| | P96B13 | 4.29 (100%) | 3.91 (91.1%) | 2.97 (69.2%) | 1.34 (31.2%) |
| ***Xlf$^{-/-}$*** | 16488 | 4.28 (100%) | 3.54 (82.7%) | 2.82 (65.9%) | 0.97 (22.7%) |
| | X95-3 | 4.3 (100%) | 3.6 (83.7%) | 2.53 (58.8%) | 0.84 (19.5%) |
| ***Paxx$^{-/-}$ Xlf$^{-/-}$*** | XP1 | 4.2 (100%) | 2.73 (65.0%) | 1.23 (29.3%) | 0.62 (14.8%) |
| | XP6 | 4.36 (100%) | 2.88 (66.1%) | 0.86 (19.7%) | 0.5 (11.5%) |
| ***Xrcc4$^{-/-}$*** | Xr-95-21 | 4.68 (100%) | 3.02 (64.5%) | 1.11 (23.7%) | 0.59 (12.6%) |
| | Xr-95-23 | 4.57 (100%) | 3.49 (76.4%) | 2.38 (52.1%) | 1.09 (23.9%) |

**B**

| | | 1 Gy | | 2.5 Gy | |
|---|---|---|---|---|---|
| | | **p value** | **Significant** | **p value** | **Significant** |
| WT | vs *Paxx$^{-/-}$* | 0.39393 | no | 0.00865 | yes |
| | vs *Xlf$^{-/-}$* | 0.01515 | yes | 0.00639 | yes |
| | vs *Paxx$^{-/-}$ Xlf$^{-/-}$* | 0.00216 | yes | 0.00216 | yes |
| | vs *Xrcc4$^{-/-}$* | 0.01515 | yes | 0.00216 | yes |
| *Paxx$^{-/-}$* | vs *Xlf$^{-/-}$* | 0.07764 | no | 0.06493 | no |
| | vs *Paxx$^{-/-}$ Xlf$^{-/-}$* | 0.00216 | yes | 0.00216 | yes |
| | vs *Xrcc4$^{-/-}$* | 0.02597 | yes | 0.00432 | yes |
| *Xlf$^{-/-}$* | vs *Paxx$^{-/-}$ Xlf$^{-/-}$* | 0.00865 | yes | 0.01515 | yes |
| | vs *Xrcc4$^{-/-}$* | 0.52110 | no | 0.30952 | no |
| *Paxx$^{-/-}$ Xlf$^{-/-}$* | vs *Xrcc4$^{-/-}$* | 0.02597 | yes | 0.09307 | no |

**Table S2. Irradiation sensitivity assay of pro-B cell lines, Related to Figure 1**
(A) Numbers indicate cell counts ($10^6$ cells/ml) after 3 days irradiation at 1Gy, 2.5Gy and 5Gy. Percentages of viable cells compared to non irradiated cells are indicated in brackets.
(B) Statistical analysis was performed at 1 and 2.5 Gy using Mann Whitney test.

# A

| EXPMT 1 | WT | | | *Paxx*$^{-/-}$ | | | *Xlf*$^{-/-}$ | | | *Paxx*$^{-/-}$ *Xlf*$^{-/-}$ | | |
|---|---|---|---|---|---|---|---|---|---|---|---|---|
| | 12095 | | | P96B1 | | | X95-3 | | | XP1 | | |
| | Nb of 53BP1 foci/nuclei | Nuclei Nb | Nuclei % | Nb of 53BP1 foci/ nuclei | Nuclei Nb | Nuclei % | Nb of 53BP1 foci/nuclei | Nuclei Nb | Nuclei % | Nb of 53BP1 foci/nuclei | Nuclei Nb | Nuclei % |
| Cell line #1 | 0 | 3387 | 67.5 | 0 | 3167 | 63 | 0 | 1879 | 37.4 | 0 | 703 | 14.1 |
| | 1 | 1236 | 24.6 | 1 | 1663 | 33.1 | 1 | 2475 | 49.3 | 1 | 2784 | 55.9 |
| | 2 | 263 | 5.2 | 2 | 167 | 3.3 | 2 | 526 | 10.5 | 2 | 1209 | 24.3 |
| | >2 | 130 | 2.6 | >2 | 33 | 0.6 | >2 | 144 | 2.8 | >2 | 284 | 5.7 |
| | TOTAL | 5016 | 100 | TOTAL | 5030 | 100 | TOTAL | 5024 | 100 | TOTAL | 4980 | 100 |
| | 12096 | | | P96B13 | | | 16488 | | | XP6 | | |
| | Nb of 53BP1 foci/nuclei | Nuclei Nb | Nuclei % | Nb of 53BP1 foci/ nuclei | Nuclei Nb | Nuclei % | Nb of 53BP1 foci/nuclei | Nuclei Nb | Nuclei % | Nb of 53BP1 foci/nuclei | Nuclei Nb | Nuclei % |
| Cell line #2 | 0 | 3204 | 63.8 | 0 | 2348 | 46.7 | 0 | 2885 | 56.7 | 0 | 721 | 14.4 |
| | 1 | 1488 | 29.6 | 1 | 2188 | 43.5 | 1 | 1727 | 33.9 | 1 | 3101 | 62 |
| | 2 | 248 | 4.9 | 2 | 400 | 8 | 2 | 360 | 7.1 | 2 | 994 | 19.9 |
| | >2 | 81 | 1.5 | >2 | 92 | 1.8 | >2 | 117 | 2.2 | >2 | 188 | 3.7 |
| | TOTAL | 5021 | 100 | TOTAL | 5028 | 100 | TOTAL | 5089 | 100 | TOTAL | 5004 | 100 |

| EXPMT 2 | WT | | | *Paxx*$^{-/-}$ | | | *Xlf*$^{-/-}$ | | | *Paxx*$^{-/-}$ *Xlf*$^{-/-}$ | | | *Xrcc4*$^{-/-}$ | | | *Ku80*$^{-/-}$ | | | *Rag2*$^{-/-}$ | | |
|---|---|---|---|---|---|---|---|---|---|---|---|---|---|---|---|---|---|---|---|---|---|
| | 12095 | | | P96B1 | | | X95-3 | | | XP1 | | | Xr-95-21 | | | 12071 | | | 129F-C1 | | |
| | Nb of 53BP1 foci/nuclei | Nuclei Nb | Nuclei % | Nb of 53BP1 foci/ nuclei | Nuclei Nb | Nuclei % | Nb of 53BP1 foci/nuclei | Nuclei Nb | Nuclei % | Nb of 53BP1 foci/nuclei | Nuclei Nb | Nuclei % | Nb of 53BP1 foci/nuclei | Nuclei Nb | Nuclei % | Nb of 53BP1 foci/nuclei | Nuclei Nb | Nuclei % | Nb of 53BP1 foci/nuclei | Nuclei Nb | Nuclei % |
| Cell line #1 | 0 | 3642 | 72.3 | 0 | 3407 | 67.9 | 0 | 3028 | 64.3 | 0 | 1373 | 27.4 | 0 | 1562 | 31.1 | 0 | 1703 | 33.6 | 0 | 3703 | 83 |
| | 1 | 958 | 19 | 1 | 1328 | 26.5 | 1 | 1292 | 27.4 | 1 | 2485 | 49.5 | 1 | 2394 | 47.6 | 1 | 2485 | 49 | 1 | 482 | 10.8 |
| | 2 | 292 | 5.8 | 2 | 200 | 4 | 2 | 292 | 6.2 | 2 | 937 | 18.7 | 2 | 864 | 17.2 | 2 | 716 | 14.1 | 2 | 166 | 3.7 |
| | >2 | 148 | 2.9 | >2 | 80 | 1.5 | >2 | 98 | 2.1 | >2 | 221 | 4.5 | >2 | 207 | 4.1 | >2 | 165 | 3,2 | >2 | 109 | 2.4 |
| | TOTAL | 5040 | 100 | TOTAL | 5015 | 100 | TOTAL | 4710 | 100 | TOTAL | 5016 | 100 | TOTAL | 5027 | 100 | TOTAL | 5069 | 100 | TOTAL | 4460 | 100 |
| | 12096 | | | P96B13 | | | 16488 | | | XP6 | | | Xr-95-23 | | | | | | | | |
| | Nb of 53BP1 foci/nuclei | Nuclei Nb | Nuclei % | Nb of 53BP1 foci/ nuclei | Nuclei Nb | Nuclei % | Nb of 53BP1 foci/nuclei | Nuclei Nb | Nuclei % | Nb of 53BP1 foci/nuclei | Nuclei Nb | Nuclei % | Nb of 53BP1 foci/nuclei | Nuclei Nb | Nuclei % | | | | | | |
| Cell line #2 | 0 | 3124 | 74.1 | 0 | 2819 | 61.5 | 0 | 3161 | 63.2 | 0 | 1495 | 29.8 | 0 | 1592 | 31.5 | | | | | | |
| | 1 | 926 | 22 | 1 | 1204 | 26.3 | 1 | 1568 | 31.3 | 1 | 2665 | 53 | 1 | 2533 | 50.1 | | | | | | |
| | 2 | 132 | 3.1 | 2 | 330 | 7.2 | 2 | 237 | 4.7 | 2 | 700 | 13.9 | 2 | 779 | 15.4 | | | | | | |
| | >2 | 36 | 0.8 | >2 | 232 | 5 | >2 | 39 | 0.7 | >2 | 164 | 3.2 | >2 | 152 | 3 | | | | | | |
| | TOTAL | 4218 | 100 | TOTAL | 4585 | 100 | TOTAL | 5005 | 100 | TOTAL | 5024 | 100 | TOTAL | 5056 | 100 | | | | | | |

| EXPMT 3 | WT | | | *Paxx*$^{-/-}$ | | | *Xlf*$^{-/-}$ | | | *Paxx*$^{-/-}$ *Xlf*$^{-/-}$ | | | *Xrcc4*$^{-/-}$ | | | *Ku80*$^{-/-}$ | | | *Rag2*$^{-/-}$ | | |
|---|---|---|---|---|---|---|---|---|---|---|---|---|---|---|---|---|---|---|---|---|---|
| | 12095 | | | P96B1 | | | X95-3 | | | XP1 | | | Xr-95-21 | | | 12071 | | | 129F-C1 | | |
| | Nb of 53BP1 foci/nuclei | Nuclei Nb | Nuclei % | Nb of 53BP1 foci/ nuclei | Nuclei Nb | Nuclei % | Nb of 53BP1 foci/nuclei | Nuclei Nb | Nuclei % | Nb of 53BP1 foci/nuclei | Nuclei Nb | Nuclei % | Nb of 53BP1 foci/nuclei | Nuclei Nb | Nuclei % | Nb of 53BP1 foci/nuclei | Nuclei Nb | Nuclei % | Nb of 53BP1 foci/nuclei | Nuclei Nb | Nuclei % |
| Cell line #1 | 0 | 3389 | 67.7 | 0 | 3127 | 62.1 | 0 | 2146 | 42.8 | 0 | 946 | 18.9 | 0 | 1025 | 20.5 | 0 | 1891 | 37.7 | 0 | 4076 | 80,8 |
| | 1 | 1352 | 27 | 1 | 1686 | 33.5 | 1 | 2291 | 45.7 | 1 | 2672 | 53.3 | 1 | 2526 | 50.5 | 1 | 2217 | 44.3 | 1 | 720 | 14.3 |
| | 2 | 205 | 4.1 | 2 | 193 | 3.8 | 2 | 466 | 9.3 | 2 | 1163 | 23.2 | 2 | 1130 | 22.6 | 2 | 739 | 14.8 | 2 | 169 | 3.4 |
| | >2 | 58 | 1.2 | >2 | 29 | 0.6 | >2 | 106 | 2.1 | >2 | 235 | 4.7 | >2 | 325 | 6.5 | >2 | 163 | 3.3 | >2 | 79 | 1.5 |
| | TOTAL | 5004 | 100 | TOTAL | 5035 | 100 | TOTAL | 5009 | 100 | TOTAL | 5016 | 100 | TOTAL | 5006 | 100 | TOTAL | 5010 | 100 | TOTAL | 5044 | 100 |
| | 12096 | | | P96B13 | | | 16488 | | | XP6 | | | Xr-95-23 | | | | | | | | |
| | Nb of 53BP1 foci/nuclei | Nuclei Nb | Nuclei % | Nb of 53BP1 foci/ nuclei | Nuclei Nb | Nuclei % | Nb of 53BP1 foci/nuclei | Nuclei Nb | Nuclei % | Nb of 53BP1 foci/nuclei | Nuclei Nb | Nuclei % | Nb of 53BP1 foci/nuclei | Nuclei Nb | Nuclei % | | | | | | |
| Cell line #2 | 0 | 3381 | 67.6 | 0 | 2106 | 41.9 | 0 | 2787 | 55.5 | 0 | 786 | 15.6 | 0 | 1156 | 23.1 | | | | | | |
| | 1 | 1447 | 28.9 | 1 | 2354 | 46.8 | 1 | 1883 | 37.5 | 1 | 3073 | 61 | 1 | 2597 | 51.8 | | | | | | |
| | 2 | 138 | 2.8 | 2 | 508 | 10.1 | 2 | 306 | 6.1 | 2 | 1022 | 20.3 | 2 | 941 | 18.8 | | | | | | |
| | >2 | 35 | 0.6 | >2 | 59 | 1.1 | >2 | 45 | 0.9 | >2 | 154 | 3.1 | >2 | 315 | 6.3 | | | | | | |
| | TOTAL | 5001 | 100 | TOTAL | 5027 | 100 | TOTAL | 5021 | 100 | TOTAL | 5035 | 100 | TOTAL | 5009 | 100 | | | | | | |

# B

| WT | | | *Paxx*$^{-/-}$ | | | *Xlf*$^{-/-}$ | | | *Paxx*$^{-/-}$ *Xlf*$^{-/-}$ | | | *Xrcc4*$^{-/-}$ | | | *Ku80*$^{-/-}$ | | | *Rag2*$^{-/-}$ | | |
|---|---|---|---|---|---|---|---|---|---|---|---|---|---|---|---|---|---|---|---|---|
| Nb of 53BP1 foci/nuclei | Nuclei Nb | Nuclei % | Nb of 53BP1 foci/nuclei | Nuclei Nb | Nuclei % | Nb of 53BP1 foci/nuclei | Nuclei Nb | Nuclei % | Nb of 53BP1 foci/nuclei | Nuclei Nb | Nuclei % | Nb of 53BP1 foci/nuclei | Nuclei Nb | Nuclei % | Nb of 53BP1 foci/nuclei | Nuclei Nb | Nuclei % | Nb of 53BP1 foci/nuclei | Nuclei Nb | Nuclei % |
| 0 | 20127 | 68.8 | 0 | 16974 | 57.2 | 0 | 15886 | 53.3 | 0 | 6024 | 20.0 | 0 | 5335 | 26.6 | 0 | 3594 | 35.7 | 0 | 7779 | 81.9 |
| 1 | 7407 | 25.2 | 1 | 10423 | 35.0 | 1 | 11236 | 37.5 | 1 | 16780 | 55.8 | 1 | 10050 | 50.0 | 1 | 4702 | 46.7 | 1 | 1202 | 12.6 |
| 2 | 1278 | 4.3 | 2 | 1798 | 6.1 | 2 | 2187 | 7.3 | 2 | 6025 | 20.1 | 2 | 3714 | 18.5 | 2 | 1455 | 14.5 | 2 | 335 | 3.6 |
| >2 | 488 | 1.6 | >2 | 525 | 1.8 | >2 | 549 | 1.8 | >2 | 1246 | 4.2 | >2 | 999 | 5.0 | >2 | 328 | 3.3 | >2 | 188 | 2.0 |
| Total nuclei | 29300 | 100 | TOTAL | 29720 | 100 | TOTAL | 29858 | 100 | TOTAL | 30075 | 100 | TOTAL | 20098 | 100 | TOTAL | 10079 | 100 | TOTAL | 9504 | 100 |

**Table S3. *Paxx*$^{-/-}$ *Xlf*$^{-/-}$ *v-abl* pro–B cell harbor increased 53BP1 DDR foci after RAG induction, Related to Figure 2**

(A) Table indicates the numbers and percentages of *v-abl* pro-B cells harboring 0, 1, 2 or >2 53BP1 foci 65h after ABLki treatment. Three independent experiments are shown.

(B) Table indicates total nuclei numbers and mean percentages of the experiments detailed in table A.

## WT

| Sequence # | 5' SE | MH | N | 3' SE | clones/total |
|---|---|---|---|---|---|
| | GGTTTTTGTTCCAGTCTGTAGCACTGTG | | | CACAGTGGTAGTACTCCACTGTCTGGCTGTACAAAAACC | |
| 1 | GGTTTTTGTTCCAGTCTGTAGCACTGTG | | | CACAGTGGTAGTACTCCACTGTCTGGCTGTACAAAAACC | 38/42 |
| 2 | GGTTTTTGTTCCAGTCTGTAGCACTGTG | | GG | CACAGTGGTAGTACTCCACTGTCTGGCTGTACAAAAACC | 1/42 |
| 3 | GGTTTTTGTTCCAGTCTGTAGCACTGTG | | AG | CACAGTGGTAGTACTCCACTGTCTGGCTGTACAAAAACC | 1/42 |
| 4 | GGTTTTTGTTCCAGTCTGTAGCACTGTG | | AC | CACAGTGGTAGTACTCCACTGTCTGGCTGTACAAAAACC | 1/42 |
| 5 | GGTTTTTGTTCCAGTCTGTAGCACTGTG | | CC | CACAGTGGTAGTACTCCACTGTCTGGCTGTACAAAAACC | 1/42 |

## *Paxx*$^{-/-}$

| Sequence # | 5' SE | MH | N | 3' SE | | clones/total |
|---|---|---|---|---|---|---|
| | GGTTTTTGTTCCAGTCTGTAGCACTGTG | | | CACAGTGGTAGTACTCCACTGTCTGGCTGTACAAAAACC | | |
| 1 | GGTTTTTGTTCCAGTCTGTAGCACTGTG | | | CACAGTGGTAGTACTCCACTGTCTGGCTGTACAAAAACC | | 83/89 |
| 2 | GGTTTTTGTTCCAGTCTGTAGCACTGTG | | AG | CACAGTGGTAGTACTCCACTGTCTGGCTGTACAAAAACC | | 1/89 |
| 3 | GGTTTTTGTTCCAGTCTGTAGCACTGTG | | CC | CACAGTGGTAGTACTCCACTGTCTGGCTGTACAAAAACC | | 1/89 |
| 4 | GGTTTTTGTTCCAGTCTGTAGCACTGTG | | GGGAT | CACAGTGGTAGTACTCCACTGTCTGGCTGTACAAAAACC | | 1/89 |
| 5 | GGTTTTTGTTCCAGTCTGTAGCACTG | | | CACAGTGGTAGTACTCCACTGTCTGGCTGTACAAAAACC | | 1/89 |
| 6 | GGTTTTTGTTCCAGTCTGTAGCACTG | | | CAGTGGTAGTACTCCACTGTCTGGCTGTACAAAAACC | | 1/89 |
| 7 | GGTTTTTGTTCCAGTCTGTAGCACTGTG | | 409 bp | (-384) | *CCACGTGTATAAGAT*<br>*ATAAGAT* | 1/89 |

## *Xlf*$^{-/-}$

| Sequence # | 5' SE | MH | N | 3' SE | clones/total |
|---|---|---|---|---|---|
| | GGTTTTTGTTCCAGTCTGTAGCACTGTG | | | CACAGTGGTAGTACTCCACTGTCTGGCTGTACAAAAACC | |
| 1 | GGTTTTTGTTCCAGTCTGTAGCACTGTG | | | CACAGTGGTAGTACTCCACTGTCTGGCTGTACAAAAACC | 29/46 |
| 2 | GGTTTTTGTTCCAGTCTGTAGCACTGTG | | CC | CACAGTGGTAGTACTCCACTGTCTGGCTGTACAAAAACC | 2/46 |
| 3 | GGTTTTTGTTCCAGTCTGTAGCACTGT | | | CACAGTGGTAGTACTCCACTGTCTGGCTGTACAAAAACC | 1/46 |
| 4 | GGTTTTTGTTCCAGTCTGTAGCACTGT | | C | CACAGTGGTAGTACTCCACTGTCTGGCTGTACAAAAACC | 1/46 |
| 5 | GGTTTTTGTTCCAGTCTGTAGCACTGTG | | CG | CAGTGGTAGTACTCCACTGTCTGGCTGTACAAAAACC | 1/46 |
| 6 | GGTTTTTGTTCCAGTCTGTAGCACTG | | CG | CACAGTGGTAGTACTCCACTGTCTGGCTGTACAAAAACC | 1/46 |
| 7 | GGTTTTTGTTCCAGTCTGTAGCACTG | | CCC | CACAGTGGTAGTACTCCACTGTCTGGCTGTACAAAAACC | 1/46 |
| 8 | GGTTTTTGTTCCAGTCTGTAGCACTGTG | | | CAGTGGTAGTACTCCACTGTCTGGCTGTACAAAAACC | 1/46 |
| 9 | GGTTTTTGTTCCAGTCTGTAGCACTGT | G | | TGGTAGTACTCCACTGTCCGGCTGTACAAAAACC | 1/46 |
| 10 | GGTTTTTGTTCCAGTCTGTAGCACTG | | CG | AGTGGTAGTACTCCACTGTCTGGCTGTACAAAAACC | 1/46 |
| 11 | GGTTTTTGTTCCAGTCTGTAGCACTG | | AG | GTGGTAGTACTCCACTGTCTGGCTGTACAAAAACC | 1/46 |
| 12 | GGTTTTTGTTCCAGTCTGTAGCAC | | | CAGTGGTAGTACTCCACTGTCTGGCTGTACAAAAACC | 1/46 |
| 13 | GGTTTTTGTTCCAGTCTGTAG | CAC | | AGTGGTAGTACTCCACTGTCTGGCTGTACAAAAACC | 3/46 |
| 14 | GGTTTTTGTTCCAGTCTGTAGCACT | GT | | AGTACTCCACTGTCTGGCTGTACAAAAACC | 1/46 |
| 15 | GGTTTTTG | | | CACAGTGGTAGTACTCCACTGTCTGGCTGTACAAAAACC | 1/46 |

***Paxx**$^{-/-}$* ***Xlf**$^{-/-}$*

| Sequence # | | 5' SE | MH | N | 3' SE | | clones/total |
|---|---|---|---|---|---|---|---|
| | | GGTTTTTGTTCCAGTCTGTAGCACTGTG | | | CACAGTGGTAGTACTCCACTGTCTGGCTGTACAAAAACC | | |
| 1 | | GGTTTTTGTTCCAGTCTGTAGCACTGTG | | | CACAGTGGTAGTACTCCACTGTCTGGCTGTACAAAAACC | | 14/88 |
| 2 | | GGTTTTTGTTCCAGTCTGTAGCACTGTG | | 804 | CACAGTGGTAGTACTCCACTGTCTGGCTGTACAAAAACC | | 1/88 |
| 3 | | GGTTTTTGTTCCAGTCTGTAGCACTG | | | CACAGTGGTAGTACTCCACTGTCTGGCTGTACAAAAACC | | 1/88 |
| 4 | | GGTTTTTGTTCCAGTCTGTAGCACTG | | | CAGTGGTAGTACTCCACTGTCTGGCTGTACAAAAACC | | 1/88 |
| 5 | | GGTTTTTGTTCCAGTCTGTAGCA | C | | ACAGTGGTAGTACTCCACTGTCTGGCTGTACAAAAACC | | 1/88 |
| 6 | | GGTTTTTGTTCCAGTCTGTAGCACTGTG | | | GGTAGTACTCCACTGTCTGGCTGTACAAAAACC | | 1/88 |
| 7 | | GGTTTTTGTTCCAGTCTGTAGCACT | | A | AGTGGTAGTACTCCACTGTCTGGCTGTACAAAAACC | | 1/88 |
| 8 | | GGTTTTTGTTCCAGTCTGTAGCACT | | | AGTGGTAGTACTCCACTGTCTGGCTGTACAAAAACC | | 1/88 |
| 9 | | GGTTTTTGTTCCAGTCTGTAGCA | C | | AGTGGTAGTACTCCACTGTCTGGCTGTACAAAAACC | | 2/88 |
| 10 | | GGTTTTTGTTCCAGTCTGTAG | CAC | | AGTGGTAGTACTCCACTGTCTGGCTGTACAAAAACC | | 12/88 |
| 11 | | GGTTTTTGTTCCAGTCTGTAGCACT | GTG | | GTAGTACTCCACTGTCTGGCTGTACAAAAACC | | 2/88 |
| 12 | | GGTTTTTGTTCCAGTCTGTAGCAC | | | GTGGTAGTACTCCACTGTCTGGCTGTACAAAAACC | | 1/88 |
| 13 | | GGTTTTTGTTCCAGTCTGTAGCACTG | | | GGTAGTACTCCACTGTCTGGCTGTACAAAAACC | | 2/88 |
| 14 | | GGTTTTTGTTCCAGTCTGTAG | CA | | GTGGTAGTACTCCACTGTCTGGCTGTACAAAAACC | | 6/88 |
| 15 | | GGTTTTTGTTCCAGTCTGTAGCACT | GT | | AGTACTCCACTGTCTGGCTGTACAAAAACC | | 1/88 |
| 16 | | GGTTTTTGTTCCAGTCTG | | C | CACAGTGGTAGTACTCCACTGTCTGGCTGTACAAAAACC | | 1/88 |
| 17 | | GGTTTTTGTTCCAGTCTGTAG | | A | TGGTAGTACTCCACTGTCTGGCTGTACAAAAACC | | 1/88 |
| 18 | | GGTTTTTGTTCCAGTCT | GT | | GGTAGTACTCCACTGTCTGGCTGTACAAAAACC | | 1/88 |
| 19 | | GGTTTTTGTTCCAGTCTG | | | GGTAGTACTCCACTGTCTGGCTGTACAAAAACC | | 2/88 |
| 20 | | GGTTTTTGTTCCAGTCTG | | C | GGTAGTACTCCACTGTCTGGCTGTACAAAAACC | | 1/88 |
| 21 | | GGTTTTTGTTCCAG | | G | AGTGGTAGTACTCCACTGTCTGGCTGTACAAAAACC | | 2/88 |
| 22 | | GGTTTTTGTTCCAGTCT | GTAG | | TACTCCACTGTCTGGCTGTACAAAAACC | | 4/88 |
| 23 | | GGTTTTTGTT | C | | ACAGTGGTAGTACTCCACTGTCTGGCTGTACAAAAACC | | 1/88 |
| 24 | | GGTTTTTGTTC | CAGT | | GGTAGTACTCCACTGTCTGGCTGTACAAAGACC | | 2/88 |
| 25 | | GGTTTTTGTTCCAGTCTGTAGCACTG | | | CTGTCTGGCTGTACAAAAACC | | 1/88 |
| 26 | | GGTTTTTGTTCCAGTCT | GTA | | CTCCACTGTCTGGCTGTACAAAAACC | | 1/88 |
| 27 | | GGTTTTTGTTCCAGTCTGTAG | CACTGT | | CTGGCTGTACAAAAACC | | 1/88 |
| 28 | | GGTTTTTGTTCCAGTCTG | | G | TCCACTGTCTGGCTGTACAAAAACC | | 1/88 |
| 29 | | GGTTTTTGTTCCAGTCTGTAG | C | | TGTCTGGCTGTACAAAAACC | | 2/88 |
| 30 | | GGTTTTTGTT | C | | TCCACTGTCTGGCTGTACAAAAACC | | 1/88 |
| 31 | | GGTTTTTGTTCCAGTCTGTA | G | | GCTGTACAAAAACC | | 1/88 |
| 32 | | GGTTTTTGTTCCAG | T | | GTCTGGCTGTACAAAAACC | | 1/88 |
| 33 | | G | G | | GTAGTACTCCACTGTCTGGCTGTACAAAAACC | | 1/88 |
| 34 | | GG | | | GTACTCCACTGTCTGGCTGTACAAAAACC | | 4/88 |
| 35 | | GGTTTTTGTTC | C | | TGGCTGTACAAAAACC | | 2/88 |
| 36 | | GGTTTTTGT | TC | | TGGCTGTACAAAAACC | | 1/88 |
| 37 | | GGTTTTTGTTCCAGTCTGTA | GCAC | | (-45) | *GCACCCGGGTA*<br>*CCGGGTA* | 1/88 |
| 38 | *GGATCTCGCGGC*<br>*GGATCT* | (-53) | | T | TGTCTGGCTGTACAAAAACC | | 1/88 |
| 39 | *GCCGACCCCGGG*<br>*GCCGACC* | (-87) | | 162 bp | CACAGTGGTAGTACTCCACTGTCTGGCTGTACAAAAACC | | 1/88 |
| 40 | | GGTTTTTGTTCCAGTCTGTAGCACTGTG | | 221 bp | (-199) | *CCTGTCTTCTTGA*<br>*TTCTTGA* | 1/88 |
| 41 | | GGTTTTTGTTCCAGTCTGTAGCACTG | TG | | (-327) | *CCTTTGCAGGCA*<br>*CAGGCA* | 1/88 |
| 42 | | GGTTTTTGTTCCAGTCTGTAGCACTGTG | | 192 bp | (-347) | *CACCTGGCGACA*<br>*CTGGCGACA* | 1/88 |
| 43 | | GGTTTTTGTTCCAGTCTGTAGCACTG | | 223 bp | (-397) | *ACCTGCAAAGGC*<br>*CTGCAAAGGC* | 1/88 |
| 44 | *AGACCTCTGGCGG*<br>*AGACCT* | (-298) | CTGGCG | | (-347) | *CCTGGCGACAGG*<br>*ACAGG* | 1/88 |
| 45 | *GTCCGCCGACACC*<br>*GTCCGCC* | (-216) | | | (-440) | *GATAGTTGTGGA*<br>*TTGTGGA* | 1/88 |

**Table S4. Signal Joints sequences from WT:DEL$^{SJ}$, *Paxx*$^{-/-}$ :DEL$^{SJ}$ , *Xlf*$^{-/-}$:DEL$^{SJ}$ and *Paxx*$^{-/-}$ *Xlf*$^{-/-}$ DEL$^{SJ}$ *v-abl* pro-B cells, Related to Figure 4**

Reference sequences for 5' and 3' signal ends generated from pMX-DEL$^{SJ}$ substrate are indicated on top. Microhomologies (MH) and N-nucleotides additions are indicated. For sequences with additions greater than 5 nucleotides, the numbers of additional bases pairs are indicated instead. When signal ends are resected further than the reference sequence, the numbers of resected nucleotides are shown in brackets along with the reference sequence flanking the resected end in italics. Frequency of unique sequence is indicated (last column). *Paxx*$^{-/-}$ *Xlf*$^{-/-}$ sequences were obtained from two independent cell lines (XP1, XP6).
